# Efficacy of auricular therapy for smoking cessation: A systematic review and network meta-analysis

**DOI:** 10.18332/tid/226712

**Published:** 2026-10-29

**Authors:** Yixuan Ou, Zhongci Zhong, Junming Xuan, Xiaoqiao Qiu, Yingqi She, Zhi Li, Lijuan Dong

**Affiliations:** 1 Department of NursingZhongshan Hospital of Traditional Chinese Medicine Affiliated to Guangzhou University of Traditional Chinese MedicineZhongshanChina; 2 Accounting School, Guangdong University of Foreign StudiesGuangzhouChina

**Keywords:** auriculotherapy, smoking cessation, network meta-analysis

## Abstract

**Introduction:**

Tobacco dependence is a chronic disease that increases the risk of multiple diseases. Auricular therapy offers a promising non-pharmacological cessation approach, yet the comparative efficacy of its specific modalities remains unclear.

**Methods:**

A systematic review and network meta-analysis of randomized controlled trials was conducted within a frequentist framework. The exposure was auricular therapy. The outcomes included exhaled carbon monoxide levels, daily cigarette consumption, smoking cessation rates, nicotine dependence and withdrawal symptoms. Eight databases were searched from inception to 5 February 2026. Two reviewers independently screened, extracted data and assessed the risk of bias. Interventions were compared using league tables of effect sizes with 95% confidence intervals and relative rankings were estimated using surface under the cumulative ranking curve probabilities.

**Results:**

Thirty-six RCTs involving 3608 participants and 14 interventions were included. Compared with placebo/minimal intervention, auricular acupressure significantly reduced exhaled carbon monoxide (mean difference, MD= -4.67; 95% CI: -6.03 – -3.30), daily cigarette consumption (MD= -6.35; 95% CI: -11.41 – -1.30), nicotine dependence (MD= -2.49; 95% CI: -4.07 – -0.91) and withdrawal symptoms (MD= -6.70; 95% CI: -9.68 – -3.71), and significantly increased smoking cessation rates (OR =4.97; 95% CI: 2.65–9.30). Body/auricular acupuncture+ aromatherapy significantly reduced daily cigarette consumption (MD= -7.64; 95% CI: -15.15 – -0.12) and nicotine dependence (MD= -2.93; 95% CI: -5.53 – -0.33). Laser auricular acupuncture showed the highest point estimate for cessation (OR=7.15; 95% CI: 3.11–16.45). No statistically significant differences were detected between auricular therapies and standard pharmacotherapy.

**Conclusions:**

Auricular acupressure was the intervention associated with significant benefits across five outcomes. While these findings suggest the potential of auricular therapies as non-pharmacological alternatives, most comparisons relied on indirect evidence and several top-ranked interventions were supported by limited data. Direct head-to-head trials are needed to confirm these observations.

## Introduction

Tobacco dependence, classified under ICD-10 code F17.2, is a chronic disease characterized by nicotine addiction^1^. It significantly increases the risk of multiple malignancies, respiratory disorders and cardiovascular diseases^2^. According to the World Health Organization (WHO) 2024 report, smoking is responsible for >8 million deaths annually worldwide, representing a profound global health burden^1^.

Smoking cessation is a pivotal strategy for mitigating tobacco-related health damage, with benefit magnitude positively correlated to earlier initiation and longer abstinence duration^3^. However, individuals who attempt to quit without assistance have success rates of only about 3%, and relapse rates remain high^4^. Although nicotine replacement therapy (NRT) is effective, it frequently induces adverse reactions including palpitations, insomnia, and dizziness^5^. This underscores the need for effective non-pharmacological alternatives.

As a non-pharmacological approach, auriculotherapy is an evidence-supported intervention for smoking cessation with a significantly lower incidence of adverse events than nicotine replacement therapy and well-documented efficacy in alleviating withdrawal symptoms and improving abstinence rates^6^.

However, its clinical application involves diverse modalities (e.g. acupressure, acupuncture) and combination regimens, which complicates treatment selection. Existing evidence syntheses provide limited guidance for choosing among these specific options. Some reviews focus on a single modality or combine all ear interventions into a broad category, obscuring their distinct effects^6,7^. Other network meta-analyses have compared major intervention classes (e.g. acupuncture, auricular acupressure and NRT) but have not differentiated or ranked the specific auricular protocols used in practice^8^. Consequently, the relative efficacy of different auricular approaches for specific treatment targets remains unclear, hindering personalized treatment planning.

Unlike previous reviews that have treated auricular therapies as a single category, the present network meta-analysis directly compares and ranks 12 specific auricular therapy protocols across five key smoking cessation outcomes. A network meta-analysis was chosen because it enables the simultaneous comparison of multiple interventions through both direct and indirect evidence, ranking treatments that have never been compared head-to-head. This approach distinguishes regimens with consistent cross-outcome benefits from those effective only for particular endpoints.

## Methods

This review followed the Preferred Reporting Items for Systematic Reviews and Meta-Analysis (PRISMA) and its extended statement for Network Meta-Analysis^9,10^. The protocol has been registered in the PROSPERO registry (CRD420251114943).

### Inclusion criteria

Studies meeting the following PICO-based criteria were included: **P**articipants, diagnosed with tobacco use disorder or nicotine dependence, regardless of gender or ethnicity; **I**ntervention, any form of auricular therapy as a monotherapy or in combination with other therapies; **C**omparison, control interventions categorized as either a minimal intervention/placebo (including wait-list, minimal health advice, sham auricular therapy) or active treatment (comprising nicotine replacement therapy, bupropion or other approved medications); and Outcomes, the study reported at least one of the following pre-specified outcomes – smoking cessation rate, daily cigarette consumption, exhaled carbon monoxide (CO) level, Fagerström test for nicotine dependence score, or self-assessed scores on the Minnesota Nicotine Withdrawal Scale (MNWS).

### Exclusion criteria

Studies were excluded if they met any of the following: 1) Non-randomized controlled trial designs (e.g. animal experiments, case reports, reviews, uncontrolled trials); 2) Full text unavailable, or data within the article were missing, erroneous, or inconsistent, preventing extraction and analysis; 3) Duplicate publications (only the version with the most complete data); 4) Study populations with comorbid major psychiatric disorders (e.g. major depressive disorder, schizophrenia) or concurrent substance addictions (other than nicotine), to avoid significant confounding; and 5) Publications in languages other than English or Chinese.

### Search strategy

Two reviewers independently searched the Cochrane Library, PubMed, Web of Science, Embase, China National Knowledge Infrastructure (CNKI), WanFang Data, VIP Information, China Biology Medicine (CBM) from inception to 5 February 2026.

The search combined Medical Subject Headings (MeSH) terms and free-text keywords. The core concepts were ‘smoking cessation/tobacco use’ and ‘auricular acupuncture/auriculotherapy’. For the smoking cessation concept, terms included, but were not limited to ‘Smoking Cessation’, ‘Cigarettes’, ‘Quitting Smoking’, and related synonyms. For the intervention concept, terms such as ‘Ear Acupuncture’, ‘Auricular Acupuncture’, and ‘Auriculotherapy’ were used. The Boolean operators AND and OR were used. The complete, reproducible search strategies for all eight databases are provided in Supplementary file Part 1.

### Study selection and data extraction

Two reviewers independently screened titles and abstracts, then full texts of potentially eligible studies. Disagreements were resolved by discussion.

Following the screening, data were independently extracted by the same reviewers using a standardized form. The extracted data included: author and publication year, sample size (for both the treatment and control groups), mean age, mean duration of smoking of participants, details of the interventions and control conditions, therapy duration, follow-up duration and all reported outcome measures. The entire study selection process followed the PRISMA guidelines and its extended statement for Network Meta-Analysis.

### Risk of bias assessment

Two reviewers independently assessed the risk of bias of all included studies using the Cochrane Risk of Bias 2 tool for randomized trials (RoB 2)^11^. The evaluation covered five domains: bias arising from the randomization process, bias due to deviations from intended interventions, bias due to missing outcome data, bias in measurement of the outcome, and bias in selection of the reported result. Each domain was rated as ‘low risk’, ‘some concerns’ or ‘high risk’. An overall risk-of-bias judgment was assigned according to RoB 2 guidance. Disagreements were resolved by consensus or with a third reviewer.

### Definition and measurement of outcomes

All outcomes assessed in this network meta-analysis were defined as follows:

- *Daily cigarette consumption* as self-reported average number of cigarettes smoked per day. A reduction indicates a decrease in smoking intensity.
- *Exhaled carbon monoxide* (CO) level (ppm) as an objective, continuous biomarker with a half-life of 4–6 hours. It directly reflects the degree of tobacco smoke exposure within the preceding 24–48 hours. A reduction in its absolute value provides an objective measure of decreased smoking intensity.
- *Smoking cessation rate* as the proportion of participants who achieved sustained abstinence. Data were extracted as reported in the original studies, including self-reported abstinence or biochemically verified abstinence (e.g. exhaled CO <6 ppm) when available. For studies reporting multiple follow-up assessments, the data from the longest available follow-up time point were used in the primary analysis. The rationale is that, within a given study, a longer follow-up assessment provides a more informative measure of the intervention’s ability to help smokers maintain abstinence. The maximum follow-up duration inevitably varies across studies, and the time point that best represents sustained abstinence was selected as available from each study, thereby aligning the conceptual definition of cessation rates across studies as closely as possible. Because smoking cessation rate is inherently time-dependent, the potential impact of differences in follow-up duration was evaluated through a subgroup analysis stratified by follow-up duration (≤8 weeks vs >8 weeks), as described in the subgroup analysis by follow-up duration section.
- *Nicotine dependence* as assessed using the Fagerström test for nicotine dependence (FTND), with higher scores indicating greater dependence.
- *Withdrawal symptom severity* as assessed using the Minnesota Nicotine Withdrawal Scale (MNWS), with higher scores indicating more severe symptoms. For withdrawal symptoms measured by the MNWS, all original publications were reviewed. Studies that explicitly reported the 9-item version or cited Hughes and Hatsukami (1986) were classified as version confirmed; for the remaining studies, use of the same 9-item core version was inferred based on the developers' statement that these nine items are the validated core symptoms. To assess whether this assumption affected the results, a sensitivity analysis using standardized mean difference (SMD) with Hedges' g correction was conducted. The results were nearly identical to those obtained with mean difference (MD) (Supplementary file Part 3), so MD was retained as the primary effect measure for its clinical interpretability.

### Statistical analysis

Analyses were conducted using Stata 17.0 within a frequentist framework, employing the network suite of commands for multivariate meta-analysis. Dichotomous outcomes were expressed as odds ratios (ORs) and continuous outcomes as mean differences (MDs), all with 95% confidence intervals (CIs). For the MNWS outcome, a sensitivity analysis using standardized mean differences (SMDs) was additionally performed to assess whether potential unobserved differences in scale versions could bias the results (Supplementary file Part 3). MD was retained as the primary effect measure for its clinical interpretability.

For each outcome, a network was constructed using arm-level data and visualized using network plots. Node size was proportional to the total number of participants per intervention; edge thickness was proportional to the number of studies per direct comparison. A random-effects consistency model was pre-specified as the primary analysis, assuming a common between-study heterogeneity (τ²) across comparisons. Under the consistency assumption, direct and indirect evidence was synthesized simultaneously.

Global, local, and loop-specific inconsistency were assessed using the design-by-treatment interaction model, node-splitting analysis, and the inconsistency factor (with 95% CI) for each closed loop, respectively. A p<0.05 indicated significant inconsistency. For outcomes with p≥0.05, the primary consistency model results are reported in the main text. For outcomes with p<0.05, inconsistency model results are presented. If significant inconsistency was detected, pre-specified sensitivity analyses were planned to identify potential sources, including the exclusion of individual studies contributing to inconsistent loops. The final model for each outcome was selected based on the consistency assumption after sensitivity analyses, where applicable.

In addition, the robustness of the primary findings was evaluated through three pre-specified sensitivity analyses: studies with a total sample size of <40 were excluded, those with an intervention duration of <4 weeks were removed, and for the MNWS outcome the network meta-analysis was repeated using standardized mean differences with Hedges' g correction instead of MD. The sample size and duration cutoffs followed recommended practices^12^.

The pooled effects were presented in league tables. Interventions were ranked using the surface under the cumulative ranking curve (SUCRA). SUCRA values range from 0% to 100%, with higher values indicating a greater likelihood of being the most effective intervention. Publication bias was assessed using comparison-adjusted funnel plots.

## Results

### Study selection and characteristics

A total of 744 records were identified, including 43 from PubMed, 124 from Web of Science, 104 from Embase, 67 from the Cochrane Library, 65 from CNKI, 143 from Wanfang, and 77 from VIP. In addition, 120 records were sourced from CBM. After removing 328 duplicates, 416 records underwent screening. After applying the exclusion criteria, 36 studies involving 3608 participants were included. The screening workflow is presented in Figure 1. These trials evaluated 14 distinct interventions: 12 specific auricular therapy regimens (both monotherapies and combinations), NRT as a standard pharmacological control, and a composite control group (namely, wait-list, minimal advice, and sham auricular procedures).

**Figure 1:**
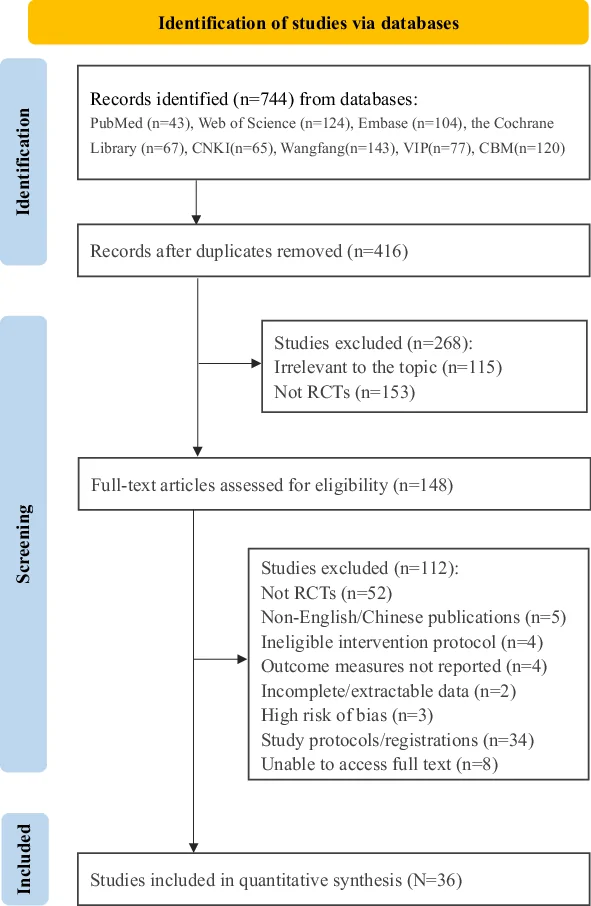
PRISMA flow diagram of study selection for a systematic review and network meta-analysis of auricular therapy for smoking cessation, based on searches of eight databases from inception to 5 February 2026.

Number of studies included in quantitative synthesis for each outcome: Exhaled CO levels, n=10; Cigarette consumption, n=15 (initial model) and n=13 (final model after sensitivity analysis for inconsistency); Smoking cessation rates, n=27; FTND scores, n=16; and MNWS scores, n=10.

Clinical heterogeneity was present across the included studies. Participants had mean ages ranging from 22 to 59 years, mean smoking durations ranging from 5 to 35 years, received interventions lasting from 1 week to 3 months, and were followed up for 2 weeks to 3 years (Supplementary file Part 2 Table 2.1). To assess the transitivity assumption, distributions of mean age and smoking duration were compared across direct comparisons (Supplementary file Part 3). Across the core comparisons, the ranges of mean age and smoking duration were broadly overlapping, with no evidence of systematic differences.

### Risks of bias

Figure 2 summarizes the risk of bias assessment for the 36 included studies^13-48^. Overall, the quality of included studies was considered low-to-moderate and 13 studies were judged as having a high risk of bias^13,15,17,20,21,24-26,28,32,38,39^^,46^.

**Figure 2:**
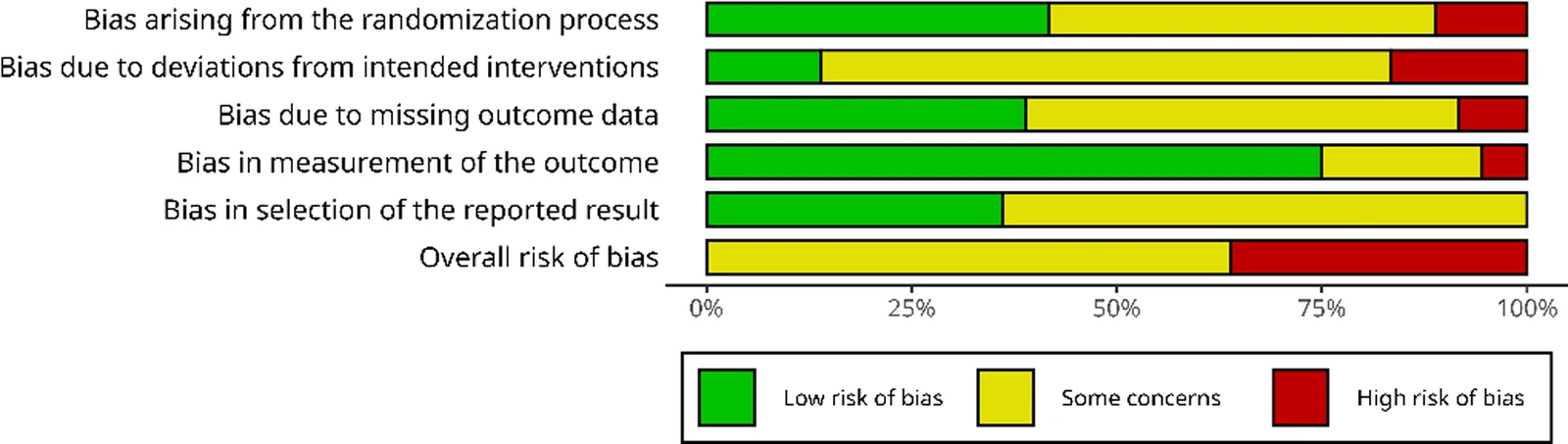
Percentages of items of included articles that produced risks of bias

Risk of bias was assessed using the Cochrane Risk of Bias 2 tool for 36 randomized controlled trials of auricular therapy for smoking cessation.

The randomization process was rated as high risk in 4 trials^17,21,26,48^, mostly due to insufficient reporting of allocation concealment or non-random allocation methods. Bias due to deviations from intended interventions was rated as high risk in 6 trials^13,15,17,26,38,39^, primarily because of open-label design without sham control or the absence of intention-to-treat analysis. Bias due to missing outcome data was rated as high risk in 3 trials^13,15,21^, mainly owing to high dropout rates or exclusion of dropouts from analysis. Bias in measurement of the outcome was rated as high risk in 2 trials^17,25^, due to reliance on unblinded self-report without biochemical verification. Bias in selection of the reported result was rated as some concerns in nearly all included studies, predominantly because of a lack of prospective trial registration.

### Network diagram

Ten studies reported exhaled CO levels^13,14,19,23,28,30,38,39,42,48^ involving 6 interventions; 15 studies reported cigarette consumption reduction^13,19-25,38,40-44,48^ involving 9 interventions; 27 studies reported smoking cessation rates^13-16,18-21,23-31,33-37,40-42,44-48^ involving 13 interventions; 16 studies reported FTND score^14,15,17-20,22-24,27-29,35,39,42,48^ involving 9 interventions; and 10 studies reported MNWS score^14,15,17,22,23,27,32,35,42,44^ involving 10 interventions. The evidence networks for each outcome are presented in Figure 3.

**Figure 3:**
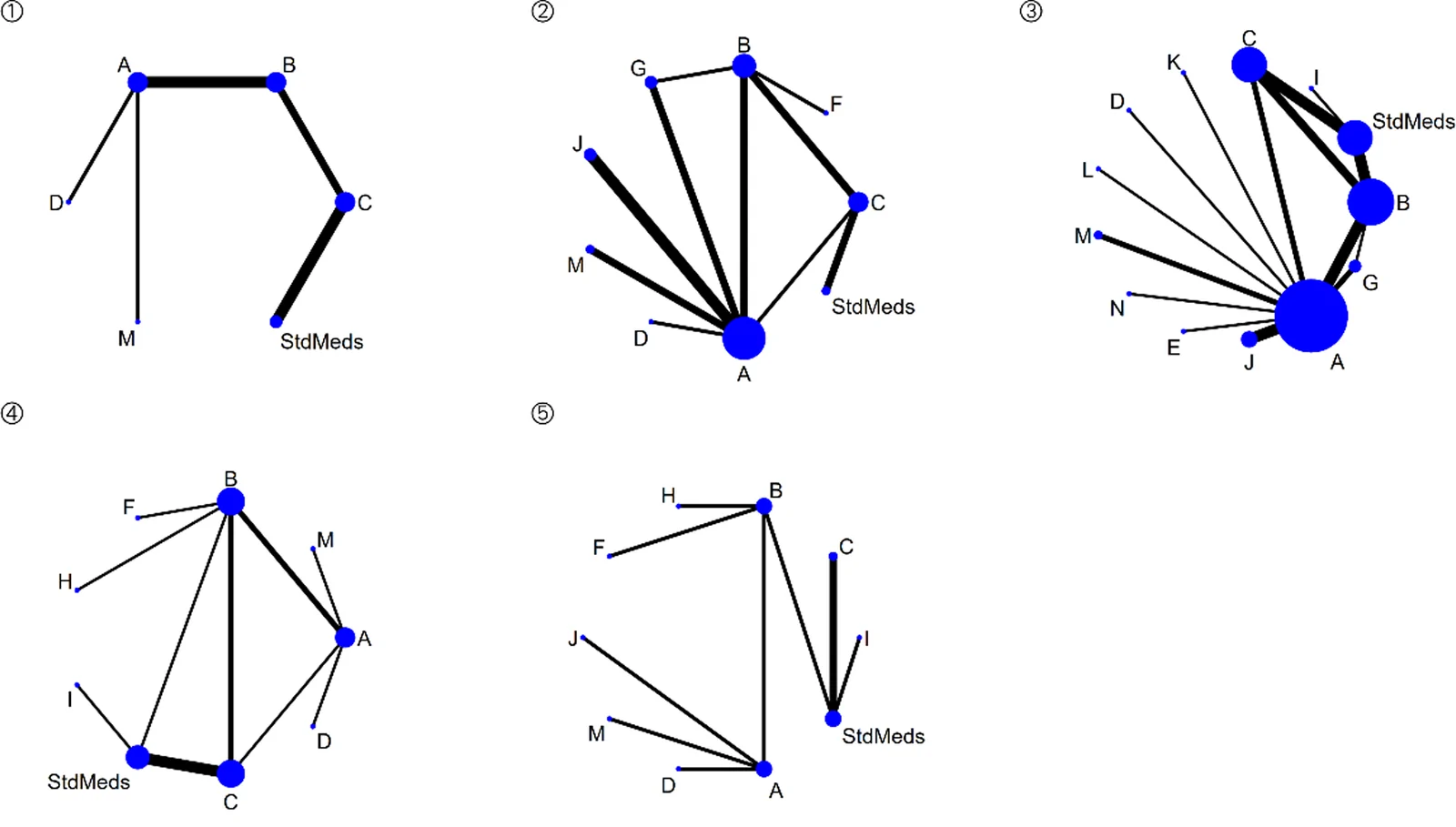
Network diagrams for five outcomes in a network meta-analysis of 14 auricular therapy interventions for smoking cessation, based on 36 randomized controlled trials. Node size reflects the number of participants per intervention and edge thickness reflects the number of studies per direct comparison

A: placebo/minimal intervention. StdMeds: standard pharmacotherapy. B: auricular acupressure. C: acupuncture + auricular acupressure. D: body/auricular acupuncture + aromatherapy. E: laser auricular acupuncture. F: auricular acupressure + transcranial direct current stimulation (tDCS). G: auricular acupressure + psychotherapy. H: auricular acupressure + Chinese herbal medicine. I: auricular acupressure + transcutaneous electrical acupoint stimulation (TEAS). J: auricular acupuncture. K: auricular electroacupuncture. L: body and auricular acupressure. M: aural electrical stimulation. N: auricular electroacupuncture + auricular acupressure. ① exhaled CO levels. ② cigarette consumption. ③ smoking cessation rates. ④ scores on the Fagerström test for nicotine dependence (FTND). ⑤ self-assessed scores on the Minnesota Nicotine Withdrawal Scale (MNWS).

### Inconsistency test

Inconsistency was assessed for cigarette consumption, smoking cessation rate, and FTND score. For cigarette consumption, the initial network (15 studies) showed significant global inconsistency (χ²=14.49, df =3, p=0.002), localized primarily to loops involving intervention G (auricular acupressure+ psychotherapy). Sensitivity analysis identified two studies^21,25^ evaluating G as the source; their exclusion resolved the inconsistency (χ²=4.60, df =3, p=0.203), thereby supporting the consistency assumption for the final model. To further assess the impact of excluding these two studies, the network structures before and after exclusion were compared. The original network contained 9 intervention nodes (Figure 3: A, B, C, D, F, G, J, M, and StdMeds) and 13 direct comparisons (edges); after exclusion, the network comprised 8 nodes (all except G) and 11 edges, with connectivity fully preserved. The SUCRA rankings shifted notably for some interventions – for example, standard pharmacotherapy moved from eighth (SUCRA=24.8%) to fourth (SUCRA=49.5%) (Supplementary file Part 5).

Global inconsistency was non-significant for smoking cessation rate (χ²=1.53, df=4, p=0.821), and FTND score (χ²=4.84, df=2, p=0.089), with no evidence of local or loop-specific inconsistency.

Inconsistency testing was not performed for exhaled CO levels and MNWS score, as no closed loops were present in these networks. Therefore, the findings for these outcomes are based solely on direct evidence, and no evidence of inconsistency exists.

### Network meta-analysis for efficacy

#### Exhaled CO levels

Compared with placebo/minimal intervention, auricular acupressure (MD= -4.67; 95% CI: -6.03 – -3.30), standard pharmacotherapy (MD= -3.98; 95% CI: -5.16 – -2.80), and aural electrical stimulation (MD= -3.39; 95% CI: -4.92 – -1.86) significantly reduced exhaled CO levels. Auricular acupressure showed a significantly greater reduction than aural electrical stimulation (MD= -1.28; 95% CI: -2.19 – -0.36) and a trend toward greater reduction compared with standard pharmacotherapy, although the difference did not reach statistical significance (MD= -0.68; 95% CI: -1.37–0.00). No significant difference was found between standard pharmacotherapy and aural electrical stimulation (MD= -0.59; 95% CI: -1.73–0.55). Acupuncture+ auricular acupressure and body/auricular acupuncture+ aromatherapy did not significantly differ from placebo (Table 1). In the SUCRA analysis, auricular acupressure had the highest probability of being the most effective intervention for reducing exhaled CO levels (99.2%), followed sequentially by standard pharmacotherapy (77.0%) and aural electrical stimulation (62.2%), while the remaining interventions were all below 50% (Supplementary file Part 4 Figure 4.1).

**Table 1:** Network meta-analysis of exhaled carbon monoxide levels in randomized controlled trials of auricular therapy for smoking cessation. Values are mean differences with 95% confidence intervals from a random-effects consistency model (10 RCTs, 6 interventions, N=541 participants)

Interventions	Placebo/minimal intervention	Standard pharmacotherapy	Auricular acupressure	Acupuncture+ auricular acupressure	Body/auricular acupuncture+ aromatherapy
Standard pharmacotherapy	-3.98 (−5.16 – -2.80)				
Auricular acupressure	-4.67 (−6.03 – -3.30)	-0.68 (−1.37–0.00)			
Acupuncture+ auricular acupressure	-0.90 (−2.79–0.99)	3.08 (0.85–5.31)	3.77 (1.43–6.10)		
Body/auricular acupuncture+ aromatherapy	2.90 (−3.08–8.88)	6.88 (0.78–12.98)	7.57 (1.43–13.70)	3.80 (−2.47–10.07)	
Aural electrical stimulation	-3.39 (−4.92 – -1.86)	0.59 (−0.55–1.73)	1.28 (0.36–2.19)	-2.49 (−4.92 – -0.06)	-6.29 (−12.46 – -0.12)

Effect estimates were obtained from a random‑effects consistency network meta‑analysis model within a frequentist framework.

#### Cigarette consumption

Compared with placebo/minimal intervention, auricular acupressure significantly reduced daily cigarette consumption (MD= -6.35; 95% CI: -11.41 – -1.30), as did body/auricular acupuncture+ aromatherapy (MD= -7.64, 95% CI: -15.15 – -0.12). Standard pharmacotherapy and the remaining interventions did not differ significantly from placebo/minimal intervention (p>0.05). Additionally, none of the auricular interventions showed statistically significant differences compared with standard pharmacotherapy (p>0.05) (Table 2).

**Table 2:** Network meta-analysis of daily cigarette consumption in randomized controlled trials of auricular therapy for smoking cessation. Values are mean differences with 95% confidence intervals from a random-effects consistency model (13 RCTs, 8 interventions, N=1063 participants)

Interventions	Placebo/minimal intervention	Standard pharmacotherapy	Auricular acupressure	Acupuncture+ auricular acupressure	Body/auricular acupuncture+ aromatherapy	Auricular acupressure+ tDCS	Auricular acupuncture
Standard pharmacotherapy	-3.31 (−8.56–1.94)						
Auricular acupressure	-6.35 (−11.41 – -1.30)	-3.04 (−7.04–0.95)					
Acupuncture+ auricular acupressure	-1.60 (−7.25–4.05)	1.71 (−6.00–9.42)	4.75 (−2.82–12.33)				
Body/auricular acupuncture+ aromatherapy	-7.64 (−15.15 – -0.12)	-4.33 (−9.71–1.05)	-1.29 (−7.98–5.41)	-6.04 (−15.44–3.36)			
Auricular acupressure+ tDCS	-0.26 (−3.75–3.23)	3.05 (−3.21–9.30)	6.09 (−0.05–12.24)	1.34 (−5.30–7.98)	7.38 (−0.87–15.62)		
Auricular acupuncture	-0.30 (−5.24–4.64)	3.01 (−4.20–10.22)	6.05 (−1.02–13.12)	1.30 (−6.21–8.80)	7.34 (−1.66–16.33)	-0.04 (−6.09–6.01)	
Aural electrical stimulation	-6.15 (−12.49–0.20)	-2.84 (−8.38–2.71)	0.21 (−3.63–4.04)	-4.55 (−13.04–3.95)	1.49 (−6.23–9.22)	-5.88 (−13.13–1.37)	-5.84 (−13.88–2.20)

Effect estimates were obtained from a random‑effects consistency network meta‑analysis model within a frequentist framework.

TEAS: electrical acupoint stimulation; tDCS: transcranial direct current stimulation.

In the SUCRA analysis, body/auricular acupuncture plus aromatherapy had the highest probability of being the most effective intervention for reducing cigarette consumption (86.2%), followed by auricular acupressure (80.4%), aural electrical stimulation (76.5%) and standard pharmacotherapy (49.5%), while other interventions were all below 40% (Supplementary file Part 4 Figure 4.2).

#### Smoking cessation rates

Compared with placebo/minimal intervention, five interventions significantly increased smoking cessation rates: standard pharmacotherapy (OR=3.55; 95% CI: 2.06–6.10), auricular acupressure (OR=4.97; 95% CI: 2.65–9.30), laser auricular acupuncture (OR=7.15; 95% CI: 3.11–16.45), auricular acupressure+ psychotherapy (OR=5.26; 95% CI: 1.52–18.29) and auricular electroacupuncture+ auricular acupressure (OR=4.53; 95% CI: 2.23–9.18). Laser auricular acupuncture had the highest point estimate (Table 3). Compared with standard pharmacotherapy, no auricular intervention demonstrated a statistically significant difference in cessation rates (Table 3).

**Table 3:** Network meta-analysis of smoking cessation rates in randomized controlled trials of auricular therapy for smoking cessation. Values are odds ratios with 95% confidence intervals from a random-effects consistency model (27 RCTs, 13 interventions, N=3311 participants)

Interventions	Placebo/minimal intervention	Standard Pharmaco- therapy	Auricular acupressure	Acupuncture+ auricular acupressure	Body/auricular acupuncture+ aromatherapy	Laser auricular acupuncture	Auricular acupressure+ psychotherapy	Auricular acupressure+ ** TEAS **	Auricular acupuncture	Auricular electroacupuncture	Body and auricular acupressure	Aural electrical stimulation
Standard pharmacotherapy	3.55 (2.06–6.10)											
Auricular acupressure	4.97 (2.65–9.30)	1.40 (0.82–2.40)										
Acupuncture+ auricular acupressure	2.18 (0.45–10.56)	0.61 (0.12–3.26)	0.44 (0.08–2.40)									
Body/auricular acupuncture+ aromatherapy	0.93 (0.33–2.59)	0.26 (0.08–0.84)	0.19 (0.06–0.62)	2.35 (0.36–15.43)								
Laser auricular acupuncture	7.15 (3.11–16.45)	2.02 (0.83–4.87)	1.44 (0.54–3.81)	3.28 (0.55–19.50)	7.70 (2.05–28.91)							
Auricular acupressure+ psychotherapy	5.26 (1.52–18.29)	1.48 (0.47–4.70)	1.06 (0.33–3.43)	2.41 (0.32–18.00)	5.67 (1.13–28.50)	0.74 (0.18–3.09)						
Auricular acupressure+ TEAS	1.78 (0.74–4.26)	0.50 (0.18–1.40)	0.36 (0.12–1.05)	0.81 (0.13–4.94)	1.91 (0.50–7.38)	0.25 (0.07–0.83)	0.34 (0.07–1.54)					
Auricular acupuncture	0.70 (0.18–2.80)	0.20 (0.04–0.87)	0.14 (0.03–0.64)	0.32 (0.04–2.62)	0.76 (0.14–4.24)	0.10 (0.02–0.49)	0.13 (0.02–0.86)	0.40 (0.08–2.03)				
Auricular electroacupuncture	2.66 (0.23–30.90)	0.75 (0.06–9.24)	0.53 (0.04–6.73)	1.22 (0.07–22.51)	2.86 (0.20–40.93)	0.37 (0.03–4.96)	0.50 (0.03–7.91)	1.50 (0.11–20.26)	3.78 (0.23–63.13)			
Body and auricular acupressure	1.35 (0.47–3.86)	0.38 (0.12–1.24)	0.27 (0.08–0.92)	0.62 (0.09–4.12)	1.45 (0.33–6.32)	0.19 (0.05–0.72)	0.26 (0.05–1.31)	0.76 (0.19–2.99)	1.92 (0.34–10.89)	0.51 (0.04–7.33)		
Aural electrical stimulation	13.52 (0.64–284.27)	3.81 (0.17–84.04)	2.72 (0.12–60.98)	6.20 (0.20–191.29)	14.57 (0.59–362.51)	1.89 (0.08–44.46)	2.57 (0.10–68.97)	7.62 (0.32–181.18)	19.23 (0.68–544.98)	5.09 (0.10–254.20)	10.02 (0.40–251.26)	
Auricular electroacupuncture+ auricular acupressure	4.53 (2.23–9.18)	1.28 (0.75–2.16)	0.91 (0.51–1.62)	2.08 (0.37–11.69)	4.88 (1.40–16.98)	0.63 (0.23–1.73)	0.86 (0.31–2.40)	2.55 (0.83–7.83)	6.44 (1.36–30.40)	1.70 (0.13–21.90)	3.36 (0.95–11.90)	0.33 (0.01, 7.63)

Effect estimates were obtained from a random‑effects consistency network meta‑analysis model within a frequentist framework.

TEAS: electrical acupoint stimulation.

In the SUCRA analysis, laser auricular acupuncture showed the highest probability of being the most effective intervention for smoking cessation (84.7%), followed by aural electrical stimulation (82.9%), auricular acupressure (74.4%), auricular acupressure+ psychotherapy (73.9%), and auricular electroacupuncture+ auricular acupressure (70.3%), while other interventions were all below 60% (Supplementary file Part 4 Figure 4.3).

#### Scores on the Fagerström test for nicotine dependence (FTND)

In reducing FTND scores, standard pharmacotherapy (MD= -1.56; 95% CI: -2.93 – -0.19), auricular acupressure (MD= -2.49; 95% CI: -4.07 – -0.91), body/auricular acupuncture+ aromatherapy (MD= -2.93; 95% CI: -5.53 – -0.33), and aural electrical stimulation (MD= -2.06; 95% CI: -3.84 – -0.28) showed statistically significant reductions compared with placebo/minimal intervention. Other interventions did not show statistically significant differences. Compared with standard pharmacotherapy, none of the interventions showed statistically significant differences (Table 4).

**Table 4:** Network meta-analysis of nicotine dependence measured by the fagerström test for nicotine dependence in randomized controlled trials of auricular therapy for smoking cessation. values are mean differences with 95% confidence intervals from a random-effects consistency model (16 RCTs, 9 interventions, N=1497 participants)

Interventions	Placebo/minimal intervention	Standard pharmacotherapy	Auricular acupressure	Acupuncture+ auricular acupressure	Body/auricular acupuncture+ aromatherapy	Auricular acupressure+ tDCS	Auricular acupressure+ chinese herbal medicine	Auricular acupressure+ ** TEAS **
Standard pharmacotherapy	-1.56 (−2.93 – -0.19)							
Auricular acupressure	-2.49 (−4.07 – -0.91)	-0.93 (−2.17–0.31)						
Acupuncture+ auricular acupressure	0.30 (−1.86–2.46)	1.86 (−0.70–4.42)	2.79 (0.11–5.47)					
Body/auricular acupuncture+ aromatherapy	-2.93 (−5.53 – -0.33)	-1.37 (−3.58–0.84)	-0.44 (−2.98–2.10)	-3.23 (−6.61–0.15)				
Auricular acupressure+ tDCS	-2.34 (−4.93–0.25)	-0.78 (−2.97–1.41)	0.15 (−2.37–2.67)	-2.64 (−6.01–0.73)	0.59 (−2.53–3.71)			
Auricular acupressure + Chinese herbal medicine	-2.56 (−5.45–0.33)	-1.00 (−3.69–1.69)	-0.07 (−2.58–2.44)	-2.86 (−6.47–0.75)	0.37 (−3.11–3.85)	-0.22 (−3.69–3.25)		
Auricular acupressure + TEAS	-0.20 (−2.87–2.47)	1.36 (−1.64–4.36)	2.29 (−0.81–5.39)	-0.50 (−2.93–3.93)	2.73 (−1.00–6.46)	2.14 (−1.57–5.85)	2.36 (−1.57–6.30)	
Aural electrical stimulation	-2.06 (−3.84 – -0.28)	-0.50 (−1.92–0.92)	0.43 (−0.62–1.48)	-2.36 (−5.16–0.44)	0.87 (−1.76–3.50)	0.28 (−2.33–2.89)	0.50 (−1.78–2.78)	-1.86 (−5.07–1.35)

Effect estimates were obtained from a random‑effects consistency network meta‑analysis model within a frequentist framework.

TEAS: electrical acupoint stimulation; tDCS: transcranial direct current stimulation.

In the SUCRA analysis, body/auricular acupuncture+ aromatherapy had the highest probability of being the most effective intervention for reducing FTND scores (SUCRA =79.1%), followed by auricular acupressure (75.4%), auricular acupressure+ Chinese herbal medicine (71.7%), auricular acupressure+ tDCS (67.0%), and aural electrical stimulation (59.7%), while the remaining interventions all had SUCRA values below 50% (Supplementary file Part 3 Figure 3.4).

#### Self-assessed scores on the Minnesota nicotine withdrawal scale (MNWS)

Compared with placebo/minimal intervention, auricular acupressure (MD= -6.70; 95% CI: -9.68 – -3.71), body/auricular acupuncture + aromatherapy (MD= -6.51; 95% CI: -8.70 – -4.32), auricular acupressure + tDCS (MD= -5.75; 95% CI: -8.59 – -2.91), aural electrical stimulation (MD= -5.37; 95% CI: -7.96 – -2.78), standard pharmacotherapy (MD= -4.28; 95% CI: -5.70 – -2.86), and auricular acupressure + Chinese herbal medicine (MD= -3.86; 95% CI: -7.25 – -0.47) had significantly better efficacy in reducing MNWS scores (Table 5).

**Table 5:** Network meta-analysis of withdrawal symptoms measured by the Minnesota nicotine withdrawal scale in randomized controlled trials of auricular therapy for smoking cessation. values are mean differences with 95% confidence intervals from a random-effects consistency model (10 RCTs, 10 interventions, N=1052 participants)

Interventions	Placebo/minimal intervention	Standard pharmacotherapy	Auricular acupressure	Acupuncture + auricular acupressure	Body/auricular acupuncture + ** aromatherapy **	Auricular acupressure + ** tDCS **	Auricular acupressure + Chinese herbal medicine	Auricular acupressure + ** TEAS **	Auricular acupuncture
Standard pharmacotherapy	-4.28 (−5.70 – -2.86)								
Auricular acupressure	-6.70 (−9.68 – -3.71)	-2.42 (−5.04–0.21)							
Acupuncture + auricular acupressure	0.50 (−3.92–4.92)	4.78 (0.14–9.42)	7.20 (1.86–12.53)						
Body/auricular acupuncture + aromatherapy	-6.51 (−8.70 – -4.32)	2.23 (0.56–3.90)	0.19 (−2.93–3.30)	-7.01 (−11.95 – -2.07)					
Auricular acupressure + tDCS	-5.75 (−8.59 – -2.91)	1.47 (−0.99–3.93)	0.95 (−2.65–4.54)	-6.25 (−11.50 – -1.00)	-0.76 (−3.73–2.21)				
Auricular acupressure + Chinese herbal medicine	-3.86 (−7.25 – -0.47)	-0.42 (−3.50–2.66)	2.84 (0.20–5.48)	-4.36 (−9.93–1.21)	-2.65 (−6.16–0.86)	-1.89 (−5.83–2.05)			
Auricular acupressure + TEAS	-0.70 (−2.12–0.72)	-3.58 (−5.59–-1.57)	6.00 (2.69–9.30)	-1.20 (−5.84–3.44)	-5.81 (−8.42 – -3.20)	-5.05 (−8.22 – -1.88)	-3.16 (−6.84–0.52)		
Auricular acupuncture	-0.60 (−4.00–2.80)	-3.68 (−7.37–0.01)	6.10 (1.57–10.62)	-1.10 (−6.68–4.48)	-5.91 (−9.96 – -1.86)	-5.15 (−9.58 – -0.72)	-3.26 (−8.07–1.55)	0.10 (−3.79–3.59)	
Aural electrical stimulation	-5.37 (−7.96 – -2.78)	1.09 (−1.08–3.26)	1.33 (−0.15–2.80)	-5.87 (−11.00 – -0.74)	-1.14 (−3.88–1.60)	-0.38 (−3.66–2.90)	1.51 (−0.68–3.70)	4.67 (1.71–7.63)	4.77 (0.49–9.05)

Effect estimates were obtained from a random‑effects consistency network meta‑analysis model within a frequentist framework.

TEAS: electrical acupoint stimulation. tDCS: transcranial direct current stimulation.

In the SUCRA analysis, auricular acupressure had the highest probability of being the most effective intervention for reducing MNWS scores (90.4%), followed by body/auricular acupuncture + aromatherapy (87.3%), auricular acupressure + tDCS (76.9%), aural electrical stimulation (70.6%), and standard pharmacotherapy (54.2%), while the remaining interventions all had SUCRA values below 50% (Supplementary file Part 4 Figure 4.5).

### Sensitivity analyses

In all three sensitivity analyses, the direction, magnitude, and statistical significance of the pooled effect estimates were similar to those from the primary analysis, and the SUCRA rankings were nearly identical. For example, after excluding studies with a total sample size of <40, auricular acupressure remained the highest-ranked intervention for exhaled carbon monoxide (SUCRA =98.8% vs 99.2% in the primary analysis) and for withdrawal symptoms (SUCRA =90.4% vs 89.7%). After excluding studies with an intervention duration of <4 weeks, the identity of the top-ranked interventions was unchanged across all outcomes. The SMD sensitivity analysis for the MNWS outcome yielded effect estimates nearly identical to those obtained with MD. Minor numerical fluctuations were observed for some interventions. The overall hierarchy of treatment efficacy and the identity of the top-ranked interventions remained unchanged. Detailed results are provided in Supplementary file Part 5.

### Subgroup analysis by follow-up duration

The results were broadly consistent with the primary analysis. In the ≤8 weeks subgroup (7 interventions), aural electrical stimulation showed the highest SUCRA value (93.8%; OR=14.98; 95% CI: 4.52–49.69) and auricular acupressure the second highest (86.7%; OR=12.28; 95% CI: 4.22–35.69), mirroring the primary analysis. In the >8 weeks subgroup (10 interventions), laser auricular acupuncture had the highest SUCRA value (82.7%), and auricular acupressure remained among the top-ranked interventions (69.1%). The absence of certain interventions (e.g. aural electrical stimulation) from the >8 weeks subgroup reflects their representation only in studies with shorter follow-up, and the differences between subgroups are therefore attributable to changes in network composition rather than systematic differences in the efficacy of individual therapies. Detailed results are provided in Supplementary file 1 Part 6.

### Publication bias

Visual inspection of funnel plots suggested asymmetry for some outcomes (Supplementary file Part 3 Figure 3.6). However, Egger’s test did not indicate statistically significant small-study effects for any outcome: exhaled CO levels (p=0.435; n=10 studies), cigarette consumption (p=0.264; n=13), smoking cessation rates (p=0.060; n=27), FTND scores (p=0.706; n=16), and MNWS scores (p=0.212; n=10) (Supplementary file Part 4 Figure 4.7).

## Discussion

This network meta-analysis, based on 36 RCTs, evaluated the comparative efficacy of 12 auricular therapy against minimal/placebo controls and nicotine replacement therapy, ranking them across five key smoking cessation outcomes. The findings provide a nuanced evidence base to inform clinical decision-making.

The network diagrams revealed several structural features of the evidence base. Taking the smoking cessation network as an example, auricular acupressure and placebo/minimal intervention formed the largest nodes, reflecting the greater number of studies comparing these interventions. The networks were generally well connected, although direct comparisons between several active interventions were absent. Across the five outcomes, the number of interventions per network ranged from 6 to 13 depending on data availability.

For objective physiological indicators, auricular acupressure had the highest SUCRA value, significantly outperforming placebo. Aural electrical stimulation also showed significant efficacy. The magnitude of CO reduction with auricular acupressure exceeds the typical minimal clinically important difference (MCID) of 2–3 ppm proposed in smoking cessation trials, supporting its clinical relevance. When directly compared with standard pharmacotherapy, auricular acupressure showed a trend toward superiority that approached statistical significance, highlighting its potential as a non-pharmacological alternative. This aligns with a previous meta-analysis that found no significant difference between broader auricular acupoint-related therapies and standard pharmacotherapy (MD=0.73; 95% CI: -0.53–1.98)^6^. However, that study aggregated all auricular acupoint-related therapies into a single category, which may have diluted the signal from the most effective individual modalities. By focusing specifically on auricular acupressure, our analysis provides greater precision.

For cigarette consumption, sensitivity analysis resolved initial network inconsistency by excluding two studies. In the final model, auricular acupressure and body/auricular acupuncture + aromatherapy significantly reduced daily cigarette consumption compared with placebo, with effect sizes exceeding the commonly suggested MCID of 3–5 cigarettes per day. Standard pharmacotherapy did not differ significantly from placebo and no other interventions reached statistical significance. Previous meta-analyses found no significant difference in daily smoking volume between auricular acupoint-related therapies and nicotine replacement therapy^6,8^. Our findings partially align with these reports in that we also detected no statistically significant difference between auricular therapies and standard pharmacotherapy. However, by disaggregating specific auricular modalities, we identified that auricular acupressure and body/auricular acupuncture plus aromatherapy were each significantly superior to placebo – a comparison that was not separately examined in the previous meta-analyses^6,8^.

Although body/auricular acupuncture + aromatherapy had the highest probability of being the most effective intervention for cigarette consumption, this finding should be interpreted cautiously given the wide confidence intervals and limited direct evidence for some comparisons. In contrast, auricular acupressure, which ranked second, was supported by multiple studies and yielded a more stable estimate. Therefore, the SUCRA ranking for this outcome should be interpreted with caution and the more robust finding is that both interventions were associated with significant reductions in cigarette consumption relative to placebo.

Regarding smoking cessation rates, five interventions showed statistically significant superiority over placebo/minimal intervention. Laser auricular acupuncture had the highest SUCRA value and point estimate, followed by auricular acupressure + psychotherapy, auricular acupressure, auricular electroacupuncture + auricular acupressure and standard pharmacotherapy. However, laser auricular acupuncture was supported by only one study and its effect estimate was imprecise, whereas auricular acupressure was supported by multiple studies and yielded a considerably more precise estimate. Thus, for smoking cessation, the fact that laser auricular acupuncture ranked first by SUCRA should not be taken as evidence that it is superior to auricular acupressure, which ranked second but rests on a more substantial body of evidence.

Furthermore, sensitivity analyses confirmed that the exclusion of small studies and short-duration interventions did not materially alter the smoking cessation findings. In addition, because cessation rates are inherently time-dependent, a subgroup analysis stratified by follow-up duration was conducted to assess whether differences in the timing of outcome assessment influenced the comparative efficacy estimates. The results were broadly consistent between the ≤8 weeks and >8 weeks subgroups, suggesting that follow-up duration did not systematically affect the findings. One exception was that body + auricular acupressure ranked low in the primary analysis but highest in the >8 weeks subgroup. This shift reflects the fact that body + auricular acupressure was supported by only one study, and its effect estimate is inherently imprecise. In the larger primary network, its signal was diluted among numerous comparisons with stronger evidence, whereas in the smaller >8 weeks network, its point estimate gained disproportionate influence on the SUCRA ranking – a pattern that is expected for interventions with sparse evidence when network size changes. The change in ranking therefore does not indicate a genuine improvement in comparative efficacy. Rather, the change illustrates the instability of SUCRA rankings for sparsely supported interventions across networks of different sizes. In direct comparisons with standard pharmacotherapy, no auricular intervention demonstrated a statistically significant difference in cessation rates, indicating that no statistically significant difference was detected between auricular therapies and standard pharmacotherapy.

For nicotine dependence measured by the FTND, four interventions showed significant benefits compared with placebo/minimal intervention: body/auricular acupuncture + aromatherapy showed the largest effect, followed by auricular acupressure, aural electrical stimulation and standard pharmacotherapy. SUCRA rankings mirrored this order (body/auricular acupuncture + aromatherapy, 79.1%; auricular acupressure, 75.4%). These results suggest that multiple approaches can effectively reduce physiological dependence, with combinations potentially offering synergistic benefits. Compared with standard pharmacotherapy for FTND, none of the auricular interventions showed statistically significant differences, consistent with Dai et al.^8^. The convergence of evidence across independent reviews suggests that auricular therapies do not differ significantly from pharmacotherapy for nicotine dependence and given their favorable safety profile, they may be considered for patients who cannot tolerate or prefer to avoid pharmacotherapy.

For withdrawal symptoms measured by the MNWS, six interventions significantly reduced scores compared to placebo. Auricular acupressure showed the largest effect size, followed by body/auricular acupuncture + aromatherapy, auricular acupressure + tDCS, aural electrical stimulation, standard pharmacotherapy and auricular acupressure + Chinese herbal medicine. SUCRA rankings also placed auricular acupressure as the most likely to be effective. These results suggest that auricular acupressure may be particularly effective for alleviating withdrawal symptoms. Given that withdrawal symptoms strongly predict relapse^49^, the consistent efficacy of auricular therapies, especially acupressure, is clinically meaningful and supports their use for patients struggling with withdrawal. This finding is consistent with a previous meta-analysis that reported auricular acupoint-related therapies significantly reduced Minnesota Nicotine Withdrawal Scale scores compared with nicotine replacement therapy (MD=1.47; 95% CI: 0.06–2.88, p<0.05)^6^. Our analysis extends this observation by identifying auricular acupressure as the specific modality most strongly associated with withdrawal symptom reduction.

Clinical heterogeneity was evident across the included studies, with participant age ranging from 22 to 59 years, smoking duration from 5 to 35 years and intervention duration from 1 week to 3 months. The transitivity assumption was assessed by comparing the distributions of mean age and smoking duration across direct comparisons, and broadly overlapping ranges were found, suggesting that these patient-level characteristics did not differ systematically across treatment comparisons. Additionally, because smoking cessation rates are inherently time-dependent, a subgroup analysis stratified by follow-up duration was conducted and did not suggest that follow-up length materially affected cessation rates. All analyses used random-effects models to account for unexplained heterogeneity. Meta-regression was not performed because several comparisons in the network were supported by only one or two studies, which can yield unstable estimates. Future reviews with a larger evidence base may benefit from such analyses.

### Limitations

This study has several limitations. First, most comparisons relied on indirect evidence because direct head-to-head trials between auricular therapies were scarce, and the limited number of studies per intervention produced wide confidence intervals for some effect estimates; consequently, SUCRA rankings should be interpreted cautiously. Second, most outcomes were self-reported and therefore subject to recall bias, and not all studies employed biochemical verification of smoking status. Third, small-study effects cannot be excluded despite non-significant Egger’s tests, particularly for outcomes with fewer than 10–15 studies. Fourth, the included studies were predominantly conducted in China, which may limit generalizability. Finally, clinical heterogeneity in intervention duration and follow-up time may have influenced the results, although this was addressed through subgroup and sensitivity analyses.

For the cigarette consumption outcome, the initial network showed significant inconsistency, which was resolved by excluding two studies. Because the exclusion was based solely on statistical inconsistency and was pre-specified in the analysis plan, it reduces concern about selective reporting bias. For the remaining outcomes, inconsistency testing did not detect significant discrepancies between direct and indirect evidence, supporting the use of the pre-specified random-effects consistency model across all primary analyses.

Despite these limitations, the transitivity assessment, inconsistency tests, and sensitivity analyses collectively support the robustness of the conclusions. The minor numerical fluctuations observed in the sensitivity analyses were expected given the removal of studies from a network with limited connectivity. For the cigarette consumption outcome, the significant global inconsistency was resolved by excluding two studies, after which the consistency assumption was satisfied (χ²=4.60, df=3, p=0.203). The consistent benefit of auricular acupressure across all five outcomes provides convergent validity that strengthens confidence in these findings, and the inclusion of 36 RCTs with 3608 participants offered robust statistical power. Future head-to-head trials are warranted to further validate these results.

## Conclusions

Among the 12 auricular therapies investigated, distinct regimens were associated with improvements in specific smoking cessation outcomes. Auricular acupressure was the only intervention associated with significant benefits across all five endpoints examined, suggesting potential cross-outcome benefits that warrant further investigation. Body/auricular acupuncture + aromatherapy also showed significant effects on cigarette consumption, nicotine dependence and withdrawal symptoms. However, several of the top-ranked interventions in these analyses were supported by only one study and their effect estimates were imprecise. Consequently, the current evidence is not yet sufficient to support a specific symptom-driven treatment algorithm, and these findings require confirmation in direct head-to-head trials before individualized treatment recommendations can be made with confidence.
