# Supplementary material for: Efficacy of auricular therapy for smoking cessation: A systematic review and network meta-analysis
Source: Tob Induc Dis. 2026 Oct 29;24:10.18332/tid/226712. doi: 10.18332/tid/226712 (PMC13720715; doi:10.18332/tid/226712)
Supplement: Supplementary file 1 [file TID-24-202-s001.docx]

**Supplementary file**

© 2026 Ou Y. et al

**DOI:** 10.18332/tid/226712

The content has been provided by the author(s) and has not been reviewed, verified, or endorsed by European Publishing. It may not have undergone peer review. The views, opinions, and recommendations expressed are solely those of the author(s) and do not necessarily reflect the position of European Publishing. European Publishing accepts no responsibility or liability for any consequences arising from the use of, or reliance on, this content.

**Supplementary File 1. Search Strategies, Transitivity Assessment** **, SUCRA Plots and Publication Bias Assessment, Sensitivity Analyses and Subgroup Analysis**

This supplementary file contains: (1) the complete search strategies for all eight databases, (2) the characteristics of the 36 included randomized controlled trials, (3) the baseline characteristics by direct comparison for transitivity assessment, (4) SUCRA plots and publication bias assessment figures moved from the main text, (5) detailed results of the sensitivity analyses and (6) the subgroup analysis stratified by follow-up duration for smoking cessation.

**Part 1. Complete Search Strategies for All Databases**

This file provides the full, reproducible search strategies executed for each of the eight databases included in this systematic review and network meta-analysis. All searches were conducted from database inception to February 5, 2026.

**1. PubMed**

**Records Retrieved: 43**

| Search number | Query | Results |
| --- | --- | --- |
| #1 | ("Tobacco Products"[Mesh]) OR "Smoking Cessation"[Mesh] | 49,999 |
| #2 | ((((((((((((((((((((((((((((((((((((((((("Smoking Cessation"[Title/Abstract]) OR ("Cessation, Smoking"[Title/Abstract])) OR ("Smoking Cessations"[Title/Abstract])) OR ("Giving Up Smoking"[Title/Abstract])) OR ("Smoking, Giving Up"[Title/Abstract])) OR ("Smokings, Giving Up"[Title/Abstract])) OR ("Up Smoking, Giving"[Title/Abstract])) OR ("Quitting Smoking"[Title/Abstract])) OR ("Smoking, Quitting"[Title/Abstract])) OR ("Stopping Smoking"[Title/Abstract])) OR ("Smoking, Stopping"[Title/Abstract])) OR ("Products, Tobacco"[Title/Abstract])) OR ("Product, Tobacco"[Title/Abstract])) OR ("Tobacco Product"[Title/Abstract])) OR ("Cigars"[Title/Abstract])) OR ("Cigar"[Title/Abstract])) OR ("Cigarettes"[Title/Abstract])) OR ("Cigarette"[Title/Abstract])) OR ("Kreteks"[Title/Abstract])) OR ("Kretek"[Title/Abstract])) OR ("Kreteks Tobacco"[Title/Abstract])) OR ("Kreteks Tobaccos"[Title/Abstract])) OR ("Tobacco, Kreteks"[Title/Abstract])) OR ("Tobaccos, Kreteks"[Title/Abstract])) OR ("Cigarillos"[Title/Abstract])) OR ("Cigarillo"[Title/Abstract])) OR ("Pipe Tobacco"[Title/Abstract])) OR ("Pipe Tobaccos"[Title/Abstract])) OR ("Tobacco, Pipe"[Title/Abstract])) OR ("Tobaccos, Pipe"[Title/Abstract])) OR ("Bidis"[Title/Abstract])) OR ("Bidi"[Title/Abstract])) OR ("Tobacco, Bidi"[Title/Abstract])) OR ("Bidi Tobacco"[Title/Abstract])) OR ("Bidi Tobaccos"[Title/Abstract])) OR ("Tobaccos, Bidi"[Title/Abstract])) OR ("Bidis Tobacco"[Title/Abstract])) OR ("Bidis Tobaccos"[Title/Abstract])) OR ("Tobacco, Bidis"[Title/Abstract])) OR ("Tobaccos, Bidis"[Title/Abstract])) OR ("Tobacco Products"[Title/Abstract]) | 123,315 |
| #3 | ("acupuncture, ear"[MeSH]) OR ("auriculotherapy"[MeSH]) | 701 |
| #4 | (((((((((("Acupunctures, Ear"[Title/Abstract]) OR ("Ear Acupunctures"[Title/Abstract])) OR ("Acupuncture, Auricular"[Title/Abstract])) OR ("Acupunctures, Auricular"[Title/Abstract])) OR ("Auricular Acupunctures"[Title/Abstract])) OR ("Auricular Acupuncture"[Title/Abstract])) OR ("Ear Acupuncture"[Title/Abstract])) OR ("Auriculotherapies"[Title/Abstract])) OR ("Acupuncture, Ear"[Title/Abstract])) OR ("Auriculotherapy"[Title/Abstract]) | 1,056 |
| #5 | #1 OR #2 | 138,708 |
| #6 | #3 OR #4 | 1,316 |
| #7 | #5 AND #6 | 43 |

**2. Web of Science**

**Records Retrieved: 124**

| Set | Query | Results |
| --- | --- | --- |
| #1 | Smoking Cessation (Topic) OR Cessation, Smoking (Topic) OR Smoking Cessations (Topic) OR Giving Up Smoking (Topic) OR Smoking, Giving Up (Topic) OR Smokings, Giving Up (Topic) OR Up Smoking, Giving (Topic) OR Quitting Smoking (Topic) OR Smoking, Quitting (Topic) OR Stopping Smoking (Topic) OR Smoking, Stopping (Topic) OR Products, Tobacco (Topic) OR Product, Tobacco (Topic) OR Tobacco Product (Topic) OR Cigars (Topic) OR Cigar (Topic) OR Cigarettes (Topic) OR Cigarette (Topic) OR Kreteks (Topic) OR Kretek (Topic) OR Kreteks Tobacco (Topic) OR Kreteks Tobaccos (Topic) OR Tobacco, Kreteks (Topic) OR Tobaccos, Kreteks (Topic) OR Cigarillos (Topic) OR Cigarillo (Topic) OR Pipe Tobacco (Topic) OR Pipe Tobaccos (Topic) OR Tobacco, Pipe (Topic) OR Tobaccos, Pipe (Topic) OR Bidis (Topic) OR Bidi (Topic) OR Tobacco, Bidi (Topic) OR Bidi Tobacco (Topic) OR Bidi Tobaccos (Topic) OR Tobaccos, Bidi (Topic) OR Bidis Tobacco (Topic) OR Bidis Tobaccos (Topic) OR Tobacco, Bidis (Topic) OR Tobaccos, Bidis (Topic) OR Tobacco Products (Topic) | 247,597 |
| #2 | Acupunctures, Ear (Topic) OR Ear Acupunctures (Topic) OR Acupuncture, Auricular (Topic) OR Acupunctures, Auricular (Topic) OR Auricular Acupunctures (Topic) OR Auricular Acupuncture (Topic) OR Ear Acupuncture (Topic) OR Auriculotherapies (Topic) OR Acupuncture, Ear (Topic) OR Auriculotherapy (Topic) | 3,139 |
| #3 | #1 AND #2 | 124 |

**3. Embase**

**Records Retrieved: 104**

| No. | Query | Results |
| --- | --- | --- |
| #1 | 'auricular acupuncture'/exp | 1,408 |
| #2 | 'smoking cessation'/exp | 80,677 |
| #3 | 'abstination, smoking':ab,ti OR 'abstinence from nicotine':ab,ti OR 'abstinence from smoking':ab,ti OR 'abstinence from tobacco':ab,ti OR 'cessation, smoking':ab,ti OR 'dehabituation, smoking':ab,ti OR 'nicotine abstination':ab,ti OR 'nicotine abstinence':ab,ti OR 'nicotine cessation':ab,ti OR 'nicotine withdrawal':ab,ti OR 'quit smoking':ab,ti OR 'smoking abstinence':ab,ti OR 'smoking dehabituation':ab,ti OR 'smoking, stopping':ab,ti OR 'stop smoking':ab,ti OR 'stopping smoking':ab,ti OR 'tobacco dehabituation':ab,ti OR 'tobacco use cessation':ab,ti OR 'smoking cessation':ab,ti | 57,647 |
| #4 | 'cigarette'/exp | 8,899 |
| #5 | 'cigarette smoking'/exp | 72,097 |
| #6 | 'cigarette smoking':ab,ti OR cigarette:ab,ti OR 'filter cigarette':ab,ti OR 'cigarette smoker':ab,ti OR 'smoking, cigarette':ab,ti | 98,414 |
| #7 | 'tobacco dependence'/exp | 30,348 |
| #8 | 'cigarette addiction':ab,ti OR 'compulsive tobacco use':ab,ti OR 'dependence, tobacco':ab,ti OR 'nicotine abuse':ab,ti OR 'nicotine addiction':ab,ti OR 'nicotine dependence':ab,ti OR 'nicotine dependency':ab,ti OR 'nicotine use disorder':ab,ti OR 'nicotinic addiction':ab,ti OR 'nicotinic dependence':ab,ti OR 'nicotinic dependency':ab,ti OR tabacism:ab,ti OR tabagism:ab,ti OR tabaquism:ab,ti OR 'tobacco abuse':ab,ti OR 'tobacco addiction':ab,ti OR 'tobacco dependency':ab,ti OR 'tobacco smoking addiction':ab,ti OR 'tobacco use disorder':ab,ti OR 'waterpipe addiction':ab,ti OR 'tobacco dependence':ab,ti | 19,522 |
| #9 | 'acupuncture, ear':ab,ti OR 'acupuncture, earlobe':ab,ti OR 'auriculo acupuncture':ab,ti OR auriculoacupuncture:ab,ti OR auriculotherapy:ab,ti OR 'ear acupuncture':ab,ti OR 'earlobe acupuncture':ab,ti OR 'auricular acupuncture':ab,ti OR 'auricular therapy':ab,ti OR 'auricular acupressure':ab,ti | 2,214 |
| #10 | #1 OR #9 | 2,685 |
| #11 | #2 OR #3 OR #4 OR #5 OR #6 OR #7 OR #8 | 224,871 |
| #12 | #10 AND #11 | 104 |

**4. Cochrane Library**

**Records Retrieved: 67**

| ID | Search | Hits |
| --- | --- | --- |
| #1 | MeSH descriptor: [Acupuncture, Ear] explode all trees | 288 |
| #2 | (Acupuncture, Ear):ti,ab,kw OR (Acupunctures, Auricular):ti,ab,kw OR (Acupuncture, Auricular):ti,ab,kw OR (Auricular Acupuncture):ti,ab,kw OR (Auricular Acupunctures):ti,ab,kw | 1,598 |
| #3 | (Ear Acupuncture):ti,ab,kw OR (Ear Acupunctures):ti,ab,kw OR (Acupunctures, Ear):ti,ab,kw OR (auricular acupressure):ti,ab,kw OR (auriculotherapy):ti,ab,kw | 1,690 |
| #4 | MeSH descriptor: [Auriculotherapy] explode all trees | 353 |
| #5 | (Auriculotherapy):ti,ab,kw OR (Auriculotherapies):ti,ab,kw OR (auricular acupuncture points):ti,ab,kw | 820 |
| #6 | #1 OR #2 OR #3 OR #4 OR #5 | 2,143 |
| #7 | MeSH descriptor: [Smoking Cessation] explode all trees | 6,035 |
| #8 | (Smoking Cessation):ti,ab,kw OR (Cessation, Smoking):ti,ab,kw OR (Smoking Cessations):ti,ab,kw OR (Giving Up Smoking):ti,ab,kw OR (Smoking, Giving Up):ti,ab,kw | 13,880 |
| #9 | (Smokings, Giving Up):ti,ab,kw OR (Up Smoking, Giving):ti,ab,kw OR (Quitting Smoking):ti,ab,kw OR (Smoking, Quitting):ti,ab,kw OR (Stopping Smoking):ti,ab,kw | 3,098 |
| #10 | (Smoking, Stopping):ti,ab,kw | 448 |
| #11 | MeSH descriptor: [Tobacco Products] explode all trees | 996 |
| #12 | (Tobacco Products):ti,ab,kw OR (Cigarillos):ti,ab,kw OR (Cigarillo):ti,ab,kw OR (Tobacco Product):ti,ab,kw OR (Product, Tobacco):ti,ab,kw | 2,291 |
| #13 | (Products, Tobacco):ti,ab,kw OR (Cigarette):ti,ab,kw OR (Cigarettes):ti,ab,kw OR (Tobacco, Bidi):ti,ab,kw OR (Bidis Tobaccos):ti,ab,kw | 11,750 |
| #14 | (Bidi Tobacco):ti,ab,kw OR (Tobacco, Bidis):ti,ab,kw OR (Tobaccos, Bidi):ti,ab,kw OR (Bidi):ti,ab,kw OR (Tobaccos, Bidis):ti,ab,kw | 52 |
| #15 | (Bidi Tobaccos):ti,ab,kw OR (Bidis):ti,ab,kw OR (Bidis Tobacco):ti,ab,kw OR (Kretek):ti,ab,kw OR (Kreteks Tobaccos):ti,ab,kw | 29 |
| #16 | (Kreteks Tobacco):ti,ab,kw OR (Kreteks):ti,ab,kw OR (Tobaccos, Kreteks):ti,ab,kw OR (Tobacco, Kreteks):ti,ab,kw OR (Cigars):ti,ab,kw | 128 |
| #17 | (Cigar):ti,ab,kw OR (Tobaccos, Pipe):ti,ab,kw OR (Pipe Tobaccos):ti,ab,kw OR (Tobacco, Pipe):ti,ab,kw OR (Pipe Tobacco):ti,ab,kw | 159 |
| #18 | #7 OR #8 OR #9 OR #10 OR #11 OR #12 OR #13 OR #14 OR #15 OR #16 OR #17 | 21,144 |
| #19 | #6 AND #18 | 67 |

**5. China National Knowledge Infrastructure (CNKI)**

**Records Retrieved: 65**

Search Query

（耳穴 + 耳穴贴压 + 耳穴压豆 + 耳穴埋豆 + 针刺耳穴 + 揿针耳穴 + 耳尖放血） AND （吸烟 + 吸烟量 + 吸烟情况 + 吸烟状况 + 吸烟者 + 戒烟 + 尼古丁依赖 + 烟草依赖）

**6. Wanfang Data**

**Records Retrieved: 143**

Search Query

(主题:(耳穴) OR 主题:(耳穴贴压) OR 主题:(耳穴压豆) OR 主题:(耳穴埋豆) OR 主题:(针刺耳穴) OR 主题:(揿针耳穴) OR 主题:(耳尖放血)) AND (主题:(吸烟) OR 主题:(吸烟量) OR 主题:(吸烟情况) OR 主题:(吸烟状况) OR 主题:(吸烟者) OR 主题:(戒烟) OR 主题:(尼古丁依赖) OR 主题:(烟草依赖))

**7. VIP Information**

**Records Retrieved: 77**

Search Query

(耳穴 OR 耳针 OR 耳压 OR 耳穴贴压 OR 耳穴埋豆 OR 耳穴疗法) AND (戒烟 OR 烟草依赖 OR 尼古丁依赖 OR 吸烟)

**8. China Biology Medicine disc (CBM / SinoMed)**

**Records Retrieved: 120**

Search Query

(("耳穴"[常用字段:智能]) OR ("耳针"[常用字段:智能]) OR ("耳压"[常用字段:智能]) OR ("耳穴贴压"[常用字段:智能]) OR ("王不留行"[常用字段:智能])) AND (("戒烟"[常用字段:智能]) OR ("烟草依赖"[常用字段:智能]) OR ("尼古丁依赖"[常用字段:智能]) OR ("吸烟"[常用字段:智能]))

**End of Part 1.**

**Part 2. Characteristics of Included Studies**

This part presents the detailed characteristics of the 36 randomized controlled trials included in this systematic review and network meta-analysis. Information on sample size, participant demographics, interventions, therapy duration, follow-up duration, and reported outcomes is provided for each study.

**Table 2.1 Characteristics of the included randomized controlled trials of auricular therapy for smoking cessation from 1 January 2000 to 5 February 2026 (N = 36).**

| Author (Year) | Sample size | | Mean age (year) | | Mean duration of smoking (year) | | Interventions | | Therapy duration | Follow-up duration | Outcome measures |
| --- | --- | --- | --- | --- | --- | --- | --- | --- | --- | --- | --- |
|  | Controls | Trials | Controls | Trials | Controls | Trials | Controls | Trials |  |  |  |
| Zhang Na 2022^13^ | 41 | 41 | 42.88±13.52 | 39.75±9.11 | 18.65±6.98 | 17.85±6.33 | Std Meds | C | 8 weeks | 8 weeks | ①②③ |
| Zhao Lin 2017^14^ | 25 | 25 | 49.32±9.04 | 45.84±7.91 | 26.90±8.977 | 28.48±7.411 | A | B | 8 weeks | 24 weeks | ①③④⑤ |
| Wu Yuan 2015^15^ | 32 | 30 | 47.57±12.67 | 47.77±12.88 | 27.0±13.6 | 27.07±10.70 | Std Meds | C | 8 weeks | 20 weeks | ②③④⑤ |
| WANG Dong-feng 2021^16^ | 80 | 80 | 36.66±4.87 | 36.66±4.87 | 15.38±4.55 | 15.15±4.63 | A | B | 3 months | 12 weeks | ③ |
| Song Rui 2023^17^ | 54 | 54 | 41.57±10.48 | 40.19±11.47 | 19.39±9.4 | 19.63±6.95 | B | H | 4 weeks | 3 months | ④⑤ |
| Peng Zijun 2015^18^ | 30 | 30 | 45.97±12.02 | 44.10±12.15 | 24.83±11.56 | 23.03±10.97 | Std Meds | C | 8 weeks | 8 weeks | ③④ |
| Luo Zhenke 2022^19^ | 30 | 30 | 38.57±8.39 | 40.23±8.87 | 19.33±7.82 | 20.53±7.74 | B | C | 8 weeks | 8 weeks | ①②③④ |
| LIU Dan 2015^20^ | 24 | 24 | 40.00±6.00 | 42.00±8.00 | 24.00±5.00 | 25.00±7.00 | B | C | 4 weeks | 4 weeks | ②③④ |
| Li Lu-bo 2011^21^ | 55 | Trials 1：57 | 46.41±11.17 | 45.75±12.21 | NR | NR | A | B | 60 days | 6 months | ②③ |
|  |  | Trials 2：63 |  | 47.66±11.71 |  | NR |  | G |  |  |  |
| KUANG H J 2021^22^ | 30 | 30 | 40.50±15.33 | 44.08±11.23 | 19.50±10.59 | 23.85±9.82 | B | F | 4 weeks | 4 weeks | ②④⑤ |
| Ji Kaisong 2023^23^ | 30 | 30 | 42.21±9.35 | 41.40±10.55 | 18.92±11.51 | 16.94±9.37 | Std Meds | C | 8 weeks | 16 weeks | ①②③④⑤ |
| Huang Ye 2012^24^ | 30 | 30 | 31.26±7.31 | 33.9±8.63 | 9.70±5.53 | 11.93±7.13 | A | C | 20 days | 3 months | ②③④ |
| GUO Bao 2020^25^ | 60 | 60 | 59.6±4.26 | 57.69±3.54 | 34.35±9.01 | 33.46±6.45 | A | G | 4 weeks | 6 months | ②③ |
| Gao Wei 2020^26^ | 50 | 52 | 42.13±9.67 | 40.26±10.03 | 23.06±7.23 | 25.26±6.08 | A | B | 30 days | 8 weeks | ③ |
| Chen Shu-Min 2022^27^ | 100 | 100 | 45.00±14.00 | 45.00±14.00 | 22.60±12.50 | 22.70±12.20 | Std Meds | I | 8 weeks | 16 weeks | ③④⑤ |
| CHEN Jiasheng 2023^28^ | 30 | 30 | 40.6±11.34 | 38.73±8.19 | NR | NR | Std Meds | C | 8 weeks | 8 weeks | ①③④ |
| Chai Xin 2019^29^ | 100 | Trials 1：100 | 45.00±14.00 | 45.00±14.00 | 22.90±12.50 | 21.50±13.50 | Std Meds | B | 8 weeks | 16 weeks | ③④ |
|  |  | Trials 2：100 |  | 46.00±13.00 |  | 21.70±12.20 |  | C |  |  |  |
| CAI Zi-bin 2022^30^ | 30 | 30 | 38.57±8.39 | 40.23±8.87 | 19.33±7.82 | 20.53±7.74 | B | C | 8 weeks | 8 weeks | ①③ |
| Cai Yiming 2000^31^ | 140 | 128 | NR | NR | NR | NR | A | E | 3 months | 3 months | ③ |
| Ta-Peng Wu 2007^32^ | 59 | 59 | 53.00±16.90 | 54.30±16.90 | 32.80±17.40 | 33.80±18.30 | A | J | 8 weeks | 6 months | ⑤ |
| Yun-Kwok Wing 2010^33^ | 38 | 32 | 46.40±11.36 | 46.50±12.36 | 28.53±11.50 | 27.11±11.39 | A | L | 3 weeks | 3 months | ③ |
| Adrian R. White 1998^34^ | 38 | 38 | 42.50±13.90 | 40.80±10.90 | 23.80±13.00 | 22.60±9.50 | A | K | 1 weeks | 2 weeks | ③ |
| Ying-ying Wang 2018^35^ | 100 | 100 | NR | NR | NR | NR | Std Meds | B | 8 weeks | 16 weeks | ③④⑤ |
| Norma R Waite 1998^36^ | 38 | 40 | 45 | 40 | 28 | 26 | A | N | 2 weeks | 6 months | ③ |
| Varaporn Sivapornpun 2024^37^ | 124 | 123 | 51.92±15.24 | 52.48±13.27 | NR | NR | Std Meds | B | 12 weeks | 24 weeks | ③ |
| Roberta de Paiva Silva 2014^38^ | 9 | 21 | NR | NR | NR | NR | A | B | 5 weeks | 9 weeks | ①② |
| Shinae Lee 2017^39^ | 26 | 27 | 22.19±2.02 | 22.67±2.02 | 62.46±27.68 (months） | 73.07±39.65 (months) | A | B | 6 weeks | 6 weeks | ①④ |
| Eun Jin Lee 2020^40^ | 32 | 42 | 25.44±2.90 | 25.62±4.72 | NR | NR | A | J | 6 weeks | 1 year | ②③ |
| Eun Jin Lee 2019^41^ | 19 | 18 | 29.42±5.84 | 29.28±9.29 | NR | NR | A | J | 6 weeks | 1 year | ②③ |
| Soobin Jang 2019^42^ | 21 | 20 | 32.9±10.26 | 37.9±12.05 | 12.9±5.47 | 16.7±9.89 | A | D | 4 weeks | 8 weeks | ①②③④⑤ |
| Dong He 2001^43^ | 20 | 26 | NR | NR | NR | NR | A | J | 3 weeks | 3 years | ② |
| Deborah J. Fritz 2013^44^ | 61 | 64 | 55.3±9.1 | 55.8±10.0 | 35.4±13.0 | 34.2±12.6 | A | M | 5 weeks | 6 weeks | ②③⑤ |
| Ian D. Bier 2002^45^ | 20 | 45 | NR | NR | NR | NR | A | J | 4 weeks | 18 weeks | ③ |
| JIANG Bin 2011^46^ | 24 | 23 | 20.63±1.91 | 20.48±1.68 | NR | NR | A | J | 3 weeks | 1 year | ③ |
| Yifan Yin 2025^47^ | 250 | 239 | 53.39±13.95 | 41.86±12.67 | NR | NR | A | C | 8 weeks | 6 months | ③ |
| Ayse Aycicegi-Dinn 2011^48^ | 23 | 24 | 34.0±10.43 | 33.8±10.26 | 16.4±9.90 | 16.1±9.64 | A | M | 3 weeks | 7 weeks | ①②③④ |

NR, Not reported; A, placebo/minimal intervention; StdMeds, Standard pharmacotherapy; B, Auricular acupressure; C, Acupuncture + auricular acupressure; D, Body/auricular acupuncture + aromatherapy; E, Laser auricular acupuncture; F, Auricular acupressure + transcranial direct current stimulation (tDCS); G, Auricular acupressure + psychotherapy; H, Auricular acupressure + Chinese herbal medicine; I, Auricular acupressure + transcutaneous electrical acupoint stimulation (TEAS); J, Auricular acupuncture; K, Auricular electroacupuncture; L, Body and auricular acupressure; M, Aural electrical stimulation; N, Auricular electroacupuncture + auricular acupressure；① Exhaled CO levels; ②Cigarette consumption；③Smoking cessation rates; ④Scores on the Fagerström Test for Nicotine Dependence (FTND); ⑤Self-assessed scores on the Minnesota Nicotine Withdrawal Scale (MNWS)

**End of Part 2**.

**Part 3. Baseline Characteristics by Direct Comparison (Transitivity Assessment)**

This part provides a summary of baseline characteristics (mean age and smoking duration) stratified by direct comparison, used to assess the transitivity assumption.

| Direct comparison | No. of studies | Mean age range (years) | Mean smoking duration range (years) |
| --- | --- | --- | --- |
| B vs. A | 6 | 22~49 | 6~28 |
| C vs. A | 2 | 31~53 | 9~11 |
| D vs. A | 1 | 32~37 | 12~16 |
| E vs. A | 1 | NR | NR |
| G vs. A | 2 | 46~59 | 33~34 |
| J vs. A | 6 | 20~54 | 32~33 |
| K vs. A | 1 | 40~43 | 23~24 |
| L vs. A | 1 | 46~47 | 27~29 |
| M vs. A | 2 | 34~56 | 16~35 |
| N vs. A | 1 | 40~45 | 26~28 |
| B vs. StdMeds | 3 | 45~52 | 21~22 |
| C vs. StdMeds | 6 | 39~48 | 17~27 |
| I vs. StdMeds | 1 | 45 | 23 |
| C vs. B | 3 | 39~42 | 19~25 |
| F vs. B | 1 | 41~44 | 20~24 |
| H vs. B | 1 | 40~42 | 19~20 |

Abbreviations: A, Placebo/minimal intervention; Std Meds, Standard pharmacotherapy; B, Auricular acupressure; C, Acupuncture + auricular acupressure; D, Body/auricular acupuncture + aromatherapy; E, Laser auricular acupuncture; F, Auricular acupressure + tDCS; G, Auricular acupressure + psychotherapy; H, Auricular acupressure + Chinese herbal medicine; I, Auricular acupressure + TEAS; J, Auricular acupuncture; K, Auricular electroacupuncture; L, Body and auricular acupressure; M, Aural electrical stimulation; N, Auricular electroacupuncture + auricular acupressure. NR, Not reported.

**End of Part 3**.

**Part 4. SUCRA Plots and Publication Bias Assessment**

This part contains the SUCRA plots and publication bias assessment figures originally presented in the main text. They have been moved to the supplementary material at the request of the reviewer.

**Fig.4.1 Surface under the cumulative ranking curve for exhaled carbon monoxide levels in a network meta-analysis of auricular therapy for smoking cessation (10 RCTs, 6 interventions).**

A, placebo/minimal intervention; StdMeds, Standard pharmacotherapy; B, auricular acupressure; C, acupuncture + auricular acupressure; D, body/auricular acupuncture + aromatherapy; M, aural electrical stimulation.

**Fig.4.2 Surface under the cumulative ranking curve for daily cigarette consumption in a network meta-analysis of auricular therapy for smoking cessation (13 RCTs, 8 interventions).**

A, placebo/minimal intervention; StdMeds, Standard pharmacotherapy; B, auricular acupressure; C, acupuncture + auricular acupressure; D, body/auricular acupuncture + aromatherapy; F, auricular acupressure + tDCS; J, auricular acupuncture; M, aural electrical stimulation.

**Fig.4.3 Surface under the cumulative ranking curve for smoking cessation rates in a network meta-analysis of auricular therapy for smoking cessation (27 RCTs, 13 interventions).**

A, placebo/minimal intervention; StdMeds, Standard pharmacotherapy; B, auricular acupressure; C, acupuncture + auricular acupressure; D, body/auricular acupuncture + aromatherapy; E, laser auricular acupuncture; G, auricular acupressure + psychotherapy; I, auricular acupressure + TEAS; J, auricular acupuncture; K, auricular electroacupuncture; L, body and auricular acupressure; M, aural electrical stimulation; N, auricular electroacupuncture + auricular acupressure

**Fig.4.4 Surface under the cumulative ranking curve for nicotine dependence (FTND) in a network meta-analysis of auricular therapy for smoking cessation (16 RCTs, 9 interventions).**

A, placebo/minimal intervention; StdMeds, Standard pharmacotherapy; B, auricular acupressure; C, acupuncture + auricular acupressure; D, body/auricular acupuncture + aromatherapy; F, auricular acupressure + tDCS; H, auricular acupressure + Chinese herbal medicine; I, auricular acupressure + TEAS; M, aural electrical stimulation.

**Fig.4.5 Surface under the cumulative ranking curve for withdrawal symptoms (MNWS) in a network meta-analysis of auricular therapy for smoking cessation (10 RCTs, 10 interventions).**

A, placebo/minimal intervention; StdMeds, Standard pharmacotherapy; B, auricular acupressure; C, acupuncture + auricular acupressure; D, body/auricular acupuncture + aromatherapy; F, auricular acupressure + tDCS; H, auricular acupressure + Chinese herbal medicine; I, auricular acupressure + TEAS; J, auricular acupuncture; M, aural electrical stimulation

**Fig.4.6 Comparison-adjusted funnel plots for publication bias assessment of five outcomes in a network meta-analysis of auricular therapy for smoking cessation, based on 36 randomized controlled trials. The red line represents the null hypothesis of no small-study effects.**

①Exhaled CO levels；②Cigarette consumption；③Quit rates；④Scores on the Fagerström Test for Nicotine Dependence (FTND)；⑤Self-assessed scores on the Minnesota Nicotine Withdrawal Scale (MNWS)

**Fig.4.7 Egger's test results for small-study effects across five outcomes in a network meta-analysis of auricular therapy for smoking cessation, based on 36 randomized controlled trials.**

①Exhaled CO levels；②Cigarette consumption；③Quit rates；④Scores on the Fagerström Test for Nicotine Dependence (FTND)；⑤Self-assessed scores on the Minnesota Nicotine Withdrawal Scale (MNWS)

**Part 5. Sensitivity Analyses**

This part presents detailed results of the sensitivity analyses conducted to evaluate the robustness of the primary network meta-analysis findings, including: (1) excluding studies with a total sample size of less than 40, (2) excluding studies with an intervention duration of less than 4 weeks, (3) comparing network structures before and after exclusion of studies causing inconsistency for the cigarette consumption outcome, and (4) using standardized mean differences (SMD) instead of mean differences (MD) for the MNWS outcome.

**1. Sensitivity Analysis by Sample Size (Total n < 40)**

**1.1 Excluded Studies**

Two studies with a total sample size of less than 40 were excluded from all analyses in this sensitivity analysis.

**Table P5.1 Studies excluded in the sample size sensitivity analysis**

| Study | Total N | Intervention comparison |
| --- | --- | --- |
| Roberta de Paiva Silva 2014 | 30 | A vs. B |
| Eun Jin Lee 2019 | 37 | A vs. J |

Abbreviations: A, Placebo/minimal intervention; B, Auricular acupressure; J, Auricular acupuncture.

**1.2 Outcomes Analyzed**

This sensitivity analysis encompassed three outcomes: exhaled CO levels, cigarette consumption, and smoking cessation rates. For each outcome, the primary analysis network and the sensitivity analysis network were re-run after excluding the two studies listed in Table P3.1. Results are presented as paired league tables and SUCRA plots below.

**1.2.1 Exhaled CO Levels**

The pooled effect estimates and SUCRA rankings after excluding studies with total N < 40 were consistent with the primary analysis.

*primary analysis (left) and sensitivity analysis (right).

**Table P5.1.1 Network Meta-analysis of exhaled CO levels** **(Primary) (MD, 95% CI)**

| Interventions | **Placebo/minimal intervention** | **Standard pharmacotherapy** | **Auricular acupressure** | **Acupuncture + auricular acupressure** | **Body/auricular acupuncture + aromatherapy** |
| --- | --- | --- | --- | --- | --- |
| **Standard pharmacotherapy** | -3.98  (-5.16, -2.80) |  |  |  |  |
| **Auricular acupressure** | -4.67  (-6.03, -3.30) | -0.68  (-1.37, 0.00) |  |  |  |
| **Acupuncture + auricular acupressure** | -0.90  (-2.79, 0.99) | 3.08  (0.85, 5.31) | 3.77  (1.43, 6.10) |  |  |
| **Body/auricular acupuncture + aromatherapy** | 2.90  (-3.08, 8.88) | 6.88  (0.78, 12.98) | 7.57  (1.43, 13.70) | 3.80  (-2.47, 10.07) |  |
| **Aural electrical stimulation** | -3.39  (-4.92, -1.86) | 0.59  (-0.55, 1.73) | 1.28  (0.36, 2.19) | -2.49  (-4.92, -0.06) | -6.29  (-12.46, -0.12) |

**Table P5.1.2 Network Meta-analysis of exhaled CO levels** **(Sensitivity) (MD, 95% CI)**

| Interventions | **Placebo/minimal intervention** | **Standard pharmacotherapy** | **Auricular acupressure** | **Acupuncture + auricular acupressure** | **Body/auricular acupuncture + aromatherapy** |
| --- | --- | --- | --- | --- | --- |
| **Standard pharmacotherapy** | -3.84  (-5.05,-2.64) |  |  |  |  |
| **Auricular acupressure** | -4.53  (-5.92,-3.14) | -0.68  (-1.37,0.00) |  |  |  |
| **Acupuncture + auricular acupressure** | -0.90  (-2.79,0.99) | 2.94  (0.70,5.19) | 3.63  (1.29,5.97) |  |  |
| **Body/auricular acupuncture + aromatherapy** | 2.90  (-3.08,8.88) | 6.74  (0.64,12.85) | 7.43  (1.29,13.57) | 3.80  (-2.47,10.07) |  |
| **Aural electrical stimulation** | -3.25  (-4.85,-1.66) | 0.59  (-0.55,1.73) | 1.27  (0.36,2.19) | -2.35  (-4.82,0.11) | -6.15  (-12.35,0.04) |

**1.2.2 Cigarette Consumption**

The pooled effect estimates and SUCRA rankings after excluding studies with total N < 40 were consistent with the primary analysis.

*primary analysis (left) and sensitivity analysis (right).

**Table P5.1.3 Network Meta-analysis of cigarette consumption** **(Primary) (MD, 95% CI)**

| Interventions | **placebo/minimal intervention** | **standard pharmacotherapy** | **auricular acupressure** | **acupuncture + auricular acupressure** | **body/auricular acupuncture + aromatherapy** | **auricular acupressure + tDCS** | **auricular acupuncture** |
| --- | --- | --- | --- | --- | --- | --- | --- |
| **Standard pharmacotherapy** | -3.31  (-8.56,1.94) |  |  |  |  |  |  |
| **auricular acupressure** | -6.35  (-11.41,-1.30) | -3.04  (-7.04,0.95) |  |  |  |  |  |
| **acupuncture + auricular acupressure** | -1.60  (-7.25,4.05) | 1.71  (-6.00,9.42) | 4.75  (-2.82,12.33) |  |  |  |  |
| **body/auricular acupuncture + aromatherapy** | -7.64  (-15.15,-0.12) | -4.33  (-9.71,1.05) | -1.29  (-7.98,5.41) | -6.04  (-15.44,3.36) |  |  |  |
| **auricular acupressure + tDCS** | -0.26  (-3.75,3.23) | 3.05  (-3.21,9.30) | 6.09  (-0.05,12.24) | 1.34  (-5.30,7.98) | 7.38  (-0.87,15.62) |  |  |
| **auricular acupuncture** | -0.30  (-5.24,4.64) | 3.01  (-4.20,10.22) | 6.05  (-1.02,13.12) | 1.30  (-6.21,8.80) | 7.34  (-1.66,16.33) | -0.04  (-6.09,6.01) |  |
| **aural electrical stimulation** | -6.15  (-12.49,0.20) | -2.84  (-8.38,2.71) | 0.21  (-3.63,4.04) | -4.55  (-13.04,3.95) | 1.49  (-6.23,9.22) | -5.88  (-13.13,1.37) | -5.84  (-13.88,2.20) |

**Table P5.1.4 Network Meta-analysis of cigarette consumption** **(Sensitivity) (MD, 95% CI)**

| Interventions | **placebo/minimal intervention** | **standard pharmacotherapy** | **auricular acupressure** | **acupuncture + auricular acupressure** | **body/auricular acupuncture + aromatherapy** | **auricular acupressure + tDCS** | **auricular acupuncture** |
| --- | --- | --- | --- | --- | --- | --- | --- |
| **Standard pharmacotherapy** | -7.12  (-14.73,0.48) |  |  |  |  |  |  |
| **auricular acupressure** | -8.96  (-15.24,-2.68) | -1.84  (-6.13,2.45) |  |  |  |  |  |
| **acupuncture + auricular acupressure** | -1.60  (-7.13,3.93) | 5.52  (-3.88,14.93) | 7.36  (-1.01,15.73) |  |  |  |  |
| **body/auricular acupuncture + aromatherapy** | -11.45  (-20.70,-2.21) | -4.33  (-9.59,0.93) | -2.49  (-9.28,4.29) | -9.85  (-20.63,0.92) |  |  |  |
| **auricular acupressure + tDCS** | -0.54  (-4.49,3.40) | 6.58  (-1.93,15.09) | 8.41  (1.00,15.83) | 1.06  (-5.74,7.85) | 10.91  (0.90,20.91) |  |  |
| **auricular acupuncture** | -0.30  (-5.18,4.57) | 6.82  (-2.22,15.85) | 8.66  (0.71,16.61) | 1.30  (-6.08,8.67) | 11.15  (0.70,21.60) | 0.24  (-6.03,6.51) |  |
| **aural electrical stimulation** | -8.75  (-16.06,-1.44) | -1.63  (-7.34,4.08) | 0.21  (-3.54,3.96) | -7.15  (-16.32,2.02) | 2.70  (-5.06,10.46) | -8.20  (-16.52,0.11) | -8.45  (-17.24,0.34) |

**1.2.3 Smoking Cessation Rates**

The pooled effect estimates and SUCRA rankings after excluding studies with total N < 40 were consistent with the primary analysis.

*primary analysis (left) and sensitivity analysis (right).

**Table P5.1.5 Network Meta-analysis of smoking cessation rates** **(Primary) (OR, 95% CI)**

| Interventions | placebo/minimal intervention | Standard pharmacotherapy | auricular acupressure | acupuncture + auricular acupressure | body/auricular acupuncture + aromatherapy | laser auricular acupuncture | auricular acupressure + psychotherapy | auricular acupressure + TEAS | auricular acupuncture | auricular electroacupuncture | body and auricular acupressure | aural electrical stimulation |
| --- | --- | --- | --- | --- | --- | --- | --- | --- | --- | --- | --- | --- |
| **Standard pharmacotherapy** | **3.55 (2.06, 6.10)** |  |  |  |  |  |  |  |  |  |  |  |
| **auricular acupressure** | **4.97 (2.65, 9.30)** | 1.40 (0.82, 2.40) |  |  |  |  |  |  |  |  |  |  |
| **acupuncture + auricular acupressure** | 2.18 (0.45, 10.56) | 0.61 (0.12, 3.26) | 0.44 (0.08, 2.40) |  |  |  |  |  |  |  |  |  |
| **body/auricular acupuncture + aromatherapy** | 0.93 (0.33, 2.59) | **0.26 (0.08, 0.84)** | **0.19 (0.06, 0.62)** | 2.35 (0.36, 15.43) |  |  |  |  |  |  |  |  |
| **laser auricular acupuncture** | **7.15 (3.11, 16.45)** | 2.02 (0.83, 4.87) | 1.44 (0.54, 3.81) | 3.28 (0.55, 19.50) | **7.70 (2.05, 28.91)** |  |  |  |  |  |  |  |
| **auricular acupressure + psychotherapy** | **5.26 (1.52, 18.29)** | 1.48 (0.47, 4.70) | 1.06 (0.33, 3.43) | 2.41 (0.32, 18.00) | **5.67 (1.13, 28.50)** | 0.74 (0.18, 3.09) |  |  |  |  |  |  |
| **auricular acupressure + TEAS** | 1.78 (0.74, 4.26) | 0.50 (0.18, 1.40) | 0.36 (0.12, 1.05) | 0.81 (0.13, 4.94) | 1.91 (0.50, 7.38) | **0.25 (0.07, 0.83)** | 0.34 (0.07, 1.54) |  |  |  |  |  |
| **auricular acupuncture** | 0.70 (0.18, 2.80) | **0.20 (0.04, 0.87)** | **0.14 (0.03, 0.64)** | 0.32 (0.04, 2.62) | 0.76 (0.14, 4.24) | **0.10 (0.02, 0.49)** | **0.13 (0.02, 0.86)** | 0.40 (0.08, 2.03) |  |  |  |  |
| **auricular electroacupuncture** | 2.66 (0.23, 30.90) | 0.75 (0.06, 9.24) | 0.53 (0.04, 6.73) | 1.22 (0.07, 22.51) | 2.86 (0.20, 40.93) | 0.37 (0.03, 4.96) | 0.50 (0.03, 7.91) | 1.50 (0.11, 20.26) | 3.78 (0.23, 63.13) |  |  |  |
| **body and auricular acupressure** | 1.35 (0.47, 3.86) | 0.38 (0.12, 1.24) | **0.27 (0.08, 0.92)** | 0.62 (0.09, 4.12) | 1.45 (0.33, 6.32) | **0.19 (0.05, 0.72)** | 0.26 (0.05, 1.31) | 0.76 (0.19, 2.99) | 1.92 (0.34, 10.89) | 0.51 (0.04, 7.33) |  |  |
| **aural electrical stimulation** | 13.52 (0.64, 284.27) | 3.81 (0.17, 84.04) | 2.72 (0.12, 60.98) | 6.20 (0.20, 191.29) | 14.57 (0.59, 362.51) | 1.89 (0.08, 44.46) | 2.57 (0.10, 68.97) | 7.62 (0.32, 181.18) | 19.23 (0.68, 544.98) | 5.09 (0.10, 254.20) | 10.02 (0.40, 251.26) |  |
| **auricular electroacupuncture + auricular acupressure** | **4.53 (2.23, 9.18)** | 1.28 (0.75, 2.16) | 0.91 (0.51, 1.62) | 2.08 (0.37, 11.69) | **4.88 (1.40, 16.98)** | 0.63 (0.23, 1.73) | 0.86 (0.31, 2.40) | 2.55 (0.83, 7.83) | **6.44 (1.36, 30.40)** | 1.70 (0.13, 21.90) | 3.36 (0.95, 11.90) | 0.33 (0.01, 7.63) |

**Table P5.1.6 Network Meta-analysis of smoking cessation rates** **(Sensitivity) (OR, 95% CI)**

| Interventions | placebo/minimal intervention | Standard pharmacotherapy | auricular acupressure | acupuncture + auricular acupressure | body/auricular acupuncture + aromatherapy | laser auricular acupuncture | auricular acupressure + psychotherapy | auricular acupressure + TEAS | auricular acupuncture | auricular electroacupuncture | body and auricular acupressure | aural electrical stimulation |
| --- | --- | --- | --- | --- | --- | --- | --- | --- | --- | --- | --- | --- |
| **Standard pharmacotherapy** | **3.55 (2.06, 6.11)** |  |  |  |  |  |  |  |  |  |  |  |
| **auricular acupressure** | **4.97 (2.65, 9.31)** | 1.40 (0.82, 2.40) |  |  |  |  |  |  |  |  |  |  |
| **acupuncture + auricular acupressure** | 2.18 (0.45, 10.57) | 0.61 (0.12, 3.26) | 0.44 (0.06, 2.80) |  |  |  |  |  |  |  |  |  |
| **body/auricular acupuncture + aromatherapy** | 0.93 (0.33, 2.59) | **0.26 (0.08, 0.84)** | **0.19 (0.06, 0.62)** | 0.43 (0.36, 15.43) |  |  |  |  |  |  |  |  |
| **laser auricular acupuncture** | **7.15 (3.11, 16.45)** | 2.02 (0.83, 4.87) | 1.44 (0.54, 3.81) | 3.28 (0.55, 19.51) | **7.70 (2.05, 28.94)** |  |  |  |  |  |  |  |
| **auricular acupressure + psychotherapy** | **5.27 (1.51, 18.31)** | 1.48 (0.47, 4.70) | 1.06 (0.33, 3.44) | 2.41 (0.32, 18.02) | **5.67 (1.13, 28.54)** | 0.74 (0.18, 3.10) |  |  |  |  |  |  |
| **auricular acupressure + TEAS** | 1.52 (0.59, 3.88) | 0.43 (0.14, 1.26) | 0.31 (0.10, 0.94) | 0.70 (0.11, 4.36) | 1.63 (0.41, 6.57) | **0.21 (0.06, 0.74)** | 0.29 (0.06, 1.37) |  |  |  |  |  |
| **auricular acupuncture** | 0.70 (0.18, 2.80) | **0.20 (0.04, 0.87)** | **0.14 (0.03, 0.64)** | 0.32 (0.04, 2.62) | 0.76 (0.14, 4.24) | **0.10 (0.02, 0.49)** | **0.13 (0.02, 0.86)** | 0.46 (0.09, 2.46) |  |  |  |  |
| **auricular electroacupuncture** | 2.66 (0.23, 30.90) | 0.75 (0.06, 9.24) | 0.53 (0.04, 6.73) | 1.22 (0.07, 22.52) | 2.86 (0.20, 40.96) | 0.37 (0.03, 4.96) | 0.50 (0.03, 7.91) | 1.75 (0.13, 24.23) | 3.78 (0.23, 63.17) |  |  |  |
| **body and auricular acupressure** | 1.35 (0.47, 3.86) | 0.38 (0.12, 1.24) | **0.27 (0.08, 0.92)** | 0.62 (0.09, 4.12) | 1.45 (0.33, 6.33) | **0.19 (0.05, 0.72)** | 0.26 (0.05, 1.31) | 0.89 (0.22, 3.64) | 1.92 (0.34, 10.90) | 0.51 (0.04, 7.33) |  |  |
| **aural electrical stimulation** | 13.52 (0.64, 284.36) | 3.81 (0.17, 84.07) | 2.72 (0.12, 60.99) | 6.20 (0.20, 191.39) | 14.57 (0.59, 362.72) | 1.89 (0.08, 44.48) | 2.57 (0.10, 69.00) | 8.91 (0.37, 215.80) | 19.23 (0.68, 545.28) | 5.09 (0.10, 254.32) | 10.02 (0.40, 251.37) |  |
| **auricular electroacupuncture + auricular acupressure** | **4.53 (2.23, 9.18)** | 1.28 (0.75, 2.16) | 0.91 (0.51, 1.62) | 2.08 (0.37, 11.70) | **4.88 (1.40, 17.00)** | 0.63 (0.23, 1.73) | 0.86 (0.31, 2.40) | 2.99 (0.92, 9.65) | **6.44 (1.36, 30.43)** | 1.70 (0.13, 21.92) | 3.36 (0.95, 11.91) | 0.33 (0.01, 7.64) |

**2. Sensitivity Analysis by Intervention Duration (< 4 Weeks)**

**2.1 Excluded Studies**

Five studies with an intervention duration of less than 4 weeks were excluded from all analyses in this sensitivity analysis.

**Table P5.2** **Studies excluded in the intervention duration sensitivity analysis**

| Study | Intervention comparison | Duration |
| --- | --- | --- |
| Huang Ye 2012 | A vs. C | 20 days |
| Yun-Kwok Wing 2010 | A vs. L | 3 weeks |
| Adrian R. White 1998 | A vs. K | 1 week |
| Norma R Waite 1998 | A vs. N | 2 weeks |
| Dong He 2001 | A vs. J | 3 weeks |
| JIANG Bin 2011 | A vs. J | 3 weeks |
| Ayse Aycicegi-Dinn 2011 | A vs. M | 3 weeks |

Abbreviations: A, Placebo/minimal intervention; C, acupuncture + auricular acupressure; J, Auricular acupuncture; K, Auricular electroacupuncture; L, Body and auricular acupressure; M, Aural electrical stimulation; N, Auricular electroacupuncture + auricular acupressure.

**2.2 Outcomes Analyzed**

This sensitivity analysis encompassed four outcomes: exhaled CO levels, cigarette consumption, smoking cessation rates, and FTND scores. For each outcome, the primary analysis network and the sensitivity analysis network were re-run after excluding the five studies listed in Table P3.2. Results are presented as paired league tables and SUCRA plots below.

**2.2.1 Exhaled CO Levels**

The pooled effect estimates and SUCRA rankings after excluding studies with duration < 4 weeks were consistent with the primary analysis.

*primary analysis (left) and sensitivity analysis (right).

**Table P5.2.1 Network Meta-analysis of exhaled CO levels** **(Primary) (MD, 95% CI)**

| Interventions | **Placebo/minimal intervention** | **Standard pharmacotherapy** | **Auricular acupressure** | **Acupuncture + auricular acupressure** | **Body/auricular acupuncture + aromatherapy** |
| --- | --- | --- | --- | --- | --- |
| **Standard pharmacotherapy** | -3.98  (-5.16, -2.80) |  |  |  |  |
| **Auricular acupressure** | -4.67  (-6.03, -3.30) | -0.68  (-1.37, 0.00) |  |  |  |
| **Acupuncture + auricular acupressure** | -0.90  (-2.79, 0.99) | 3.08  (0.85, 5.31) | 3.77  (1.43, 6.10) |  |  |
| **Body/auricular acupuncture + aromatherapy** | 2.90  (-3.08, 8.88) | 6.88  (0.78, 12.98) | 7.57  (1.43, 13.70) | 3.80  (-2.47, 10.07) |  |
| **Aural electrical stimulation** | -3.39  (-4.92, -1.86) | 0.59  (-0.55, 1.73) | 1.28  (0.36, 2.19) | -2.49  (-4.92, -0.06) | -6.29  (-12.46, -0.12) |

**Table P5.2.2 Network Meta-analysis of exhaled CO levels** **(Sensitivity) (MD, 95% CI)**

| Interventions | **Placebo/minimal intervention** | **Standard pharmacotherapy** | **Auricular acupressure** | **Acupuncture + auricular acupressure** |
| --- | --- | --- | --- | --- |
| **Standard pharmacotherapy** | -3.98  (-5.16,-2.80) |  |  |  |
| **Auricular acupressure** | -4.67  (-6.03,-3.30) | -0.68  (-1.37,0.00) |  |  |
| **Acupuncture + auricular acupressure** | -0.90  (-2.79,0.99) | 3.08  (0.85,5.31) | 3.77  (1.43,6.10) |  |
| **Body/auricular acupuncture + aromatherapy** | -3.39  (-4.92,-1.86) | 0.59  (-0.55,1.73) | 1.28  (0.36,2.19) | -2.49  (-4.92,-0.06) |

**2.2.2 Cigarette Consumption**

The pooled effect estimates and SUCRA rankings after excluding studies with duration < 4 weeks were consistent with the primary analysis.

*primary analysis (left) and sensitivity analysis (right).

**Table P5.2.3 Network Meta-analysis of cigarette consumption** **(Primary) (MD, 95% CI)**

| Interventions | **placebo/minimal intervention** | **standard pharmacotherapy** | **auricular acupressure** | **acupuncture + auricular acupressure** | **body/auricular acupuncture + aromatherapy** | **auricular acupressure + tDCS** | **auricular acupuncture** |
| --- | --- | --- | --- | --- | --- | --- | --- |
| **Standard pharmacotherapy** | -3.31  (-8.56,1.94) |  |  |  |  |  |  |
| **auricular acupressure** | -6.35  (-11.41,-1.30) | -3.04  (-7.04,0.95) |  |  |  |  |  |
| **acupuncture + auricular acupressure** | -1.60  (-7.25,4.05) | 1.71  (-6.00,9.42) | 4.75  (-2.82,12.33) |  |  |  |  |
| **body/auricular acupuncture + aromatherapy** | -7.64  (-15.15,-0.12) | -4.33  (-9.71,1.05) | -1.29  (-7.98,5.41) | -6.04  (-15.44,3.36) |  |  |  |
| **auricular acupressure + tDCS** | -0.26  (-3.75,3.23) | 3.05  (-3.21,9.30) | 6.09  (-0.05,12.24) | 1.34  (-5.30,7.98) | 7.38  (-0.87,15.62) |  |  |
| **auricular acupuncture** | -0.30  (-5.24,4.64) | 3.01  (-4.20,10.22) | 6.05  (-1.02,13.12) | 1.30  (-6.21,8.80) | 7.34  (-1.66,16.33) | -0.04  (-6.09,6.01) |  |
| **aural electrical stimulation** | -6.15  (-12.49,0.20) | -2.84  (-8.38,2.71) | 0.21  (-3.63,4.04) | -4.55  (-13.04,3.95) | 1.49  (-6.23,9.22) | -5.88  (-13.13,1.37) | -5.84  (-13.88,2.20) |

**Table P5.2.4 Network Meta-analysis of cigarette consumption** **(Sensitivity) (MD, 95% CI)**

| Interventions | **placebo/minimal intervention** | **standard pharmacotherapy** | **auricular acupressure** | **acupuncture + auricular acupressure** | **body/auricular acupuncture + aromatherapy** | **auricular acupressure + tDCS** | **auricular acupuncture** |
| --- | --- | --- | --- | --- | --- | --- | --- |
| **Standard pharmacotherapy** | -0.10  (-5.35,5.15) |  |  |  |  |  |  |
| **auricular acupressure** | -1.16  (-7.44,5.12) | -1.06  (-4.51,2.39) |  |  |  |  |  |
| **acupuncture + auricular acupressure** | -1.60  (-4.66,1.46) | -1.50  (-7.58,4.58) | -0.44  (-7.43,6.55) |  |  |  |  |
| **body/auricular acupuncture + aromatherapy** | -4.43  (-10.26,1.40) | -4.33  (-6.86,-1.80) | -3.27  (-7.54,1.01) | -2.83  (-9.41,3.75) |  |  |  |
| **auricular acupressure + tDCS** | 2.15  (-0.93,5.22) | 2.25  (-3.84,8.33) | 3.31  (-3.49,10.11) | 3.75  (-0.59,8.08) | 6.58  (-0.01,13.16) |  |  |
| **auricular acupuncture** | -0.40  (-5.38,4.58) | -0.30  (-7.54,6.94) | 0.76  (-7.25,8.78) | 1.20  (-4.64,7.04) | 4.03  (-3.63,11.69) | -2.55  (-8.39,3.30) |  |
| **aural electrical stimulation** | -0.79  (-7.67,6.09) | -0.69  (-5.13,3.75) | 0.37  (-1.52,2.26) | 0.81  (-6.72,8.34) | 3.64  (-1.47,8.75) | -2.93  (-10.22,4.35) | -0.39  (-8.88,8.10) |

**2.2.3 Smoking Cessation Rates**

The pooled effect estimates and SUCRA rankings after excluding studies with duration < 4 weeks were consistent with the primary analysis.

*primary analysis (left) and sensitivity analysis (right).

**Table P5.2.5 Network Meta-analysis of smoking cessation rates** **(Primary) (OR, 95% CI)**

| Interventions | placebo/minimal intervention | Standard pharmacotherapy | auricular acupressure | acupuncture + auricular acupressure | body/auricular acupuncture + aromatherapy | laser auricular acupuncture | auricular acupressure + psychotherapy | auricular acupressure + TEAS | auricular acupuncture | auricular electroacupuncture | body and auricular acupressure | aural electrical stimulation |
| --- | --- | --- | --- | --- | --- | --- | --- | --- | --- | --- | --- | --- |
| **Standard pharmacotherapy** | **3.55 (2.06, 6.10)** |  |  |  |  |  |  |  |  |  |  |  |
| **auricular acupressure** | **4.97 (2.65, 9.30)** | 1.40 (0.82, 2.40) |  |  |  |  |  |  |  |  |  |  |
| **acupuncture + auricular acupressure** | 2.18 (0.45, 10.56) | 0.61 (0.12, 3.26) | 0.44 (0.08, 2.40) |  |  |  |  |  |  |  |  |  |
| **body/auricular acupuncture + aromatherapy** | 0.93 (0.33, 2.59) | **0.26 (0.08, 0.84)** | **0.19 (0.06, 0.62)** | 2.35 (0.36, 15.43) |  |  |  |  |  |  |  |  |
| **laser auricular acupuncture** | **7.15 (3.11, 16.45)** | 2.02 (0.83, 4.87) | 1.44 (0.54, 3.81) | 3.28 (0.55, 19.50) | **7.70 (2.05, 28.91)** |  |  |  |  |  |  |  |
| **auricular acupressure + psychotherapy** | **5.26 (1.52, 18.29)** | 1.48 (0.47, 4.70) | 1.06 (0.33, 3.43) | 2.41 (0.32, 18.00) | **5.67 (1.13, 28.50)** | 0.74 (0.18, 3.09) |  |  |  |  |  |  |
| **auricular acupressure + TEAS** | 1.78 (0.74, 4.26) | 0.50 (0.18, 1.40) | 0.36 (0.12, 1.05) | 0.81 (0.13, 4.94) | 1.91 (0.50, 7.38) | **0.25 (0.07, 0.83)** | 0.34 (0.07, 1.54) |  |  |  |  |  |
| **auricular acupuncture** | 0.70 (0.18, 2.80) | **0.20 (0.04, 0.87)** | **0.14 (0.03, 0.64)** | 0.32 (0.04, 2.62) | 0.76 (0.14, 4.24) | **0.10 (0.02, 0.49)** | **0.13 (0.02, 0.86)** | 0.40 (0.08, 2.03) |  |  |  |  |
| **auricular electroacupuncture** | 2.66 (0.23, 30.90) | 0.75 (0.06, 9.24) | 0.53 (0.04, 6.73) | 1.22 (0.07, 22.51) | 2.86 (0.20, 40.93) | 0.37 (0.03, 4.96) | 0.50 (0.03, 7.91) | 1.50 (0.11, 20.26) | 3.78 (0.23, 63.13) |  |  |  |
| **body and auricular acupressure** | 1.35 (0.47, 3.86) | 0.38 (0.12, 1.24) | **0.27 (0.08, 0.92)** | 0.62 (0.09, 4.12) | 1.45 (0.33, 6.32) | **0.19 (0.05, 0.72)** | 0.26 (0.05, 1.31) | 0.76 (0.19, 2.99) | 1.92 (0.34, 10.89) | 0.51 (0.04, 7.33) |  |  |
| **aural electrical stimulation** | 13.52 (0.64, 284.27) | 3.81 (0.17, 84.04) | 2.72 (0.12, 60.98) | 6.20 (0.20, 191.29) | 14.57 (0.59, 362.51) | 1.89 (0.08, 44.46) | 2.57 (0.10, 68.97) | 7.62 (0.32, 181.18) | 19.23 (0.68, 544.98) | 5.09 (0.10, 254.20) | 10.02 (0.40, 251.26) |  |
| **auricular electroacupuncture + auricular acupressure** | **4.53 (2.23, 9.18)** | 1.28 (0.75, 2.16) | 0.91 (0.51, 1.62) | 2.08 (0.37, 11.69) | **4.88 (1.40, 16.98)** | 0.63 (0.23, 1.73) | 0.86 (0.31, 2.40) | 2.55 (0.83, 7.83) | **6.44 (1.36, 30.40)** | 1.70 (0.13, 21.90) | 3.36 (0.95, 11.90) | 0.33 (0.01, 7.63) |

**Table P5.2.6 Network Meta-analysis of smoking cessation rates** **(Sensitivity) (OR, 95% CI)**

| Interventions | placebo/minimal intervention | Standard pharmacotherapy | auricular acupressure | acupuncture + auricular acupressure | body/auricular acupuncture + aromatherapy | laser auricular acupuncture | auricular acupressure + psychotherapy | auricular acupressure + TEAS | auricular acupuncture |
| --- | --- | --- | --- | --- | --- | --- | --- | --- | --- |
| **Standard pharmacotherapy** | **3.67**  **(2.03,6.63)** |  |  |  |  |  |  |  |  |
| **auricular acupressure** | **5.32**  **(2.58,11.00)** | 1.45  (0.81,2.60) |  |  |  |  |  |  |  |
| **acupuncture + auricular acupressure** | 2.18  (0.43,11.09) | 0.59  (0.11,3.35) | 0.41  (0.07,2.43) |  |  |  |  |  |  |
| **body/auricular acupuncture + aromatherapy** | 0.93  (0.31,2.79) | **0.25**  **(0.07,0.88)** | **0.17**  **(0.05,0.65)** | 0.43  (0.06,3.03) |  |  |  |  |  |
| **laser auricular acupuncture** | **7.23**  **(3.01,17.35)** | 1.97  (0.77,5.01) | 1.36  (0.47,3.89) | 3.31  (0.52,20.99) | **7.79**  **(1.91,31.78)** |  |  |  |  |
| **auricular acupressure + psychotherapy** | **5.60**  **(1.46,21.47)** | 1.53  (0.45,5.23) | 1.05  (0.30,3.68) | 2.57  (0.31,21.14) | **6.03**  **(1.06,34.26)** | 0.77  (0.17,3.59) |  |  |  |
| **auricular acupressure + TEAS** | 1.64  (0.56,4.79) | 0.45  (0.13,1.52) | 0.31  (0.08,1.12) | 0.75  (0.11,5.27) | 1.77  (0.38,8.21) | **0.23**  **(0.06,0.91)** | 0.29  (0.05,1.63) |  |  |
| **auricular acupuncture** | 1.30  (0.36,4.70) | 0.35  (0.09,1.46) | 0.24 (0.06,1.07) | 0.60  (0.08,4.73) | 1.40  (0.26,7.60) | **0.18**  **(0.04,0.85)** | 0.23  (0.04,1.49) | 0.79  (0.15,4.21) |  |
| **aural electrical stimulation** | 4.81  (2.22,10.45) | 1.31  (0.75,2.29) | 0.90  (0.49,1.66) | 2.21  (0.36,13.36) | 5.19  (1.35,19.92) | 0.67  (0.23,1.94) | 0.86  (0.29,2.58) | 2.93  (0.79,10.91) | 3.70  (0.83,16.60) |

**2.2.4 FTND Scores**

The pooled effect estimates and SUCRA rankings after excluding studies with duration < 4 weeks were consistent with the primary analysis.

*primary analysis (left) and sensitivity analysis (right).

**Table P5.2.7 Network Meta-analysis of FTND** **(Primary) (MD, 95% CI)**

| Interventions | Placebo/minimal intervention | Standard pharmaco-therapy | auricular acupressure | acupuncture + auricular acupressure | body/auricular acupuncture + aromatherapy | auricular acupressure + tDCS | auricular acupressure + Chinese herbal medicine | auricular acupressure + TEAS |
| --- | --- | --- | --- | --- | --- | --- | --- | --- |
| **Standard pharmacotherapy** | -1.56  (-2.93, -0.19) |  |  |  |  |  |  |  |
| **auricular acupressure** | -2.49  (-4.07, -0.91) | -0.93  (-2.17, 0.31) |  |  |  |  |  |  |
| **acupuncture + auricular acupressure** | 0.30  (-1.86, 2.46) | 1.86  (-0.70, 4.42) | 2.79  (0.11, 5.47) |  |  |  |  |  |
| **body/auricular acupuncture + aromatherapy** | -2.93  (-5.53, -0.33) | -1.37  (-3.58, 0.84) | -0.44  (-2.98, 2.10) | -3.23  (-6.61, 0.15) |  |  |  |  |
| **auricular acupressure + tDCS** | -2.34  (-4.93, 0.25) | -0.78  (-2.97, 1.41) | 0.15 (-2.37, 2.67) | -2.64  (-6.01, 0.73) | 0.59  (-2.53, 3.71) |  |  |  |
| **auricular acupressure + Chinese herbal medicine** | -2.56  (-5.45, 0.33) | -1.00  (-3.69, 1.69) | -0.07  (-2.58, 2.44) | -2.86  (-6.47, 0.75) | 0.37  (-3.11, 3.85) | -0.22  (-3.69, 3.25) |  |  |
| **auricular acupressure + TEAS** | -0.20  (-2.87, 2.47) | 1.36  (-1.64, 4.36) | 2.29  (-0.81, 5.39) | -0.50  (-2.93, 3.93) | 2.73  (-1.00, 6.46) | 2.14  (-1.57, 5.85) | 2.36  (-1.57, 6.30) |  |
| **aural electrical stimulation** | -2.06  (-3.84, -0.28) | -0.50  (-1.92, 0.92) | 0.43  (-0.62, 1.48) | -2.36  (-5.16, 0.44) | 0.87  (-1.76, 3.50) | 0.28  (-2.33, 2.89) | 0.50  (-1.78, 2.78) | -1.86  (-5.07, 1.35) |

**Table P5.2.8 Network Meta-analysis of FTND** **(Sensitivity) (MD, 95% CI)**

| Interventions | Placebo/minimal intervention | Standard pharmaco-therapy | auricular acupressure | acupuncture + auricular acupressure | body/auricular acupuncture + aromatherapy | auricular acupressure + tDCS | auricular acupressure + Chinese herbal medicine |
| --- | --- | --- | --- | --- | --- | --- | --- |
| **Standard pharmacotherapy** | -1.00  (-2.43,0.44) |  |  |  |  |  |  |
| **auricular acupressure** | -1.48  (-3.38,0.41) | -0.49  (-1.73,0.76) |  |  |  |  |  |
| **acupuncture + auricular acupressure** | 0.30  (-1.64,2.24) | 1.30  (-1.12,3.71) | 1.78  (-0.93,4.50) |  |  |  |  |
| **body/auricular acupuncture + aromatherapy** | -2.37  (-4.83,0.10) | -1.37  (-3.38,0.64) | -0.88  (-3.24,1.48) | -2.67  (-5.80,0.47) |  |  |  |
| **auricular acupressure + tDCS** | -1.78  (-4.22,0.67) | -0.78  (-2.76,1.20) | -0.29  (-2.63,2.05) | -2.08  (-5.20,1.05) | 0.59  (-2.23,3.41) |  |  |
| **auricular acupressure + Chinese herbal medicine** | -1.65  (-4.51,1.22) | -0.65  (-3.13,1.83) | -0.16  (-2.45,2.13) | -1.95  (-5.41,1.52) | 0.72  (-2.47,3.91) | 0.13  (-3.05,3.31) |  |
| **auricular acupressure + TEAS** | -1.15  (-3.12,0.83) | -0.15  (-1.51,1.21) | 0.34  (-0.63,1.30) | -1.45  (-4.22,1.32) | 1.22  (-1.20,3.64) | 0.63  (-1.77,3.04) | 0.50  (-1.58,2.58) |

**3. Sensitivity Analysis for Cigarette Consumption: Network Structure and SUCRA Rankings Before and After Excluding Intervention G**

Two studies (Li Lu‑bo 2011 and GUO Bao 2020) involving intervention G were excluded due to significant global inconsistency.

**Table P5.3** **Studies excluded in the intervention duration sensitivity analysis**

| Parameter | Before exclusion | After exclusion |
| --- | --- | --- |
| Number of studies | 15 | 13 |
| Number of intervention nodes | 9 (A, B, C, D, F, G, J, M, StdMeds) | 8 (all except G) |
| Number of direct comparisons | 13 | 11 |
| Removed studies | — | Li Lu‑bo 2011; GUO Bao 2020 |
| Global inconsistency P‑value | 0.002 | 0.203 |

*Network plots for cigarette consumption outcome. (Left: before exclusion; Right: after exclusion)

* SUCRA Rankings Before and After Resolving Inconsistency. (Left: before exclusion; Right: after exclusion)

**4. Sensitivity Analysis for MNWS Using Standardized Mean Differences (SMD)**

To assess whether potential differences in MNWS versions across studies could affect the results, the MNWS outcome was re‑analyzed using standardized mean differences (SMD) with Hedges' g correction. The SUCRA rankings from the SMD analysis were nearly identical to those obtained with MD, confirming the robustness of the findings.

*primary analysis (left) and sensitivity analysis (right).

**End of Part 5**.

**Part 6. Subgroup Analysis by Follow-up Duration (Smoking Cessation)**

This subgroup analysis evaluates whether follow-up duration influences the comparative efficacy estimates for smoking cessation. Studies were stratified into two subgroups: ≤8 weeks and >8 weeks, based on the median follow-up duration across the included studies.

**1 ≤8 Weeks Subgroup**

This subgroup included 7 interventions (A, Std Meds, B, C, D, K, and M). The SUCRA rankings were consistent with the primary analysis: aural electrical stimulation (M) ranked highest (SUCRA = 93.8%), followed by auricular acupressure (B, SUCRA = 86.7%) and standard pharmacotherapy (Std Meds, SUCRA = 67.7%).

* SUCRA Rankings for the ≤8 weeks subgroup.

**Table P6.1** **League table for the ≤8 weeks subgroup (OR, 95% CI)**

| Interventions | placebo/minimal intervention | Standard pharmacotherapy | auricular acupressure | acupuncture + auricular acupressure | body/auricular acupuncture + aromatherapy | auricular electroacupuncture |
| --- | --- | --- | --- | --- | --- | --- |
| **Standard pharmacotherapy** | **7.78**  **(3.21,18.83)** |  |  |  |  |  |
| **auricular acupressure** | **12.28**  **(4.22,35.69)** | 1.58  (0.87,2.87) |  |  |  |  |
| **acupuncture + auricular acupressure** | 2.18  (0.57,8.41) | 0.28  (0.06,1.41) | 0.18  (0.03,0.99) |  |  |  |
| **body/auricular acupuncture + aromatherapy** | 0.70  (0.23,2.14) | **0.09**  **(0.02,0.38)** | **0.06 (0.01,0.27)** | 0.32  (0.06,1.86) |  |  |
| **auricular electroacupuncture** | 1.34  (0.59,3.03) | 0.17  (0.05,0.57) | 0.11 (0.03,0.42) | 0.61 (0.13,2.97) | 1.90 (0.48,7.58) |  |
| **aural electrical stimulation** | 14.98  (4.52,49.69) | 1.93  (0.86,4.33) | **1.22 (0.59,2.53)** | 6.87 (1.13,41.76) | 1 21.30 (4.14,109.54) | 11.22 (2.62,47.96) |

**2 >8 Weeks Subgroup**

This subgroup included 10 interventions (A, Std Meds, B, C, E, G, I, J, L, and N). Laser auricular acupuncture (E, SUCRA = 82.7%) and body and auricular acupressure (L, SUCRA = 82.5%) ranked highest, while auricular acupressure (B, SUCRA = 69.1%) remained among the top-ranked interventions.

* SUCRA Rankings for the **>**8 weeks subgroup.

**Table P6.2** **League table for the >8 weeks subgroup (OR, 95% CI)**

| Interventions | placebo/minimal intervention | Standard pharmacotherapy | auricular acupressure | acupuncture + auricular acupressure | laser auricular acupuncture | auricular acupressure + psychotherapy | auricular acupressure + TEAS | auricular acupuncture | body and auricular acupressure | aural electrical stimulation |
| --- | --- | --- | --- | --- | --- | --- | --- | --- | --- | --- |
| **Standard pharmacotherapy** | **2.87**  **(1.43,5.79)** |  |  |  |  |  |  |  |  |  |
| **auricular acupressure** | **4.47**  **(2.01,9.94)** | 1.56  (0.64,3.81) |  |  |  |  |  |  |  |  |
| **acupuncture + auricular acupressure** | 0.93  (0.31,2.79) | 0.32  (0.09,1.19) | 0.21  (0.05,0.81) |  |  |  |  |  |  |  |
| **laser auricular acupuncture** | **6.64**  **(2.73,16.11)** | 2.31  (0.88,6.06) | 1.48 (0.47,4.66) | 7.15  (1.74,29.43) |  |  |  |  |  |  |
| **auricular acupressure + psychotherapy** | **3.85**  **(0.95,15.64)** | 1.34  (0.37,4.83) | 0.86 (0.21,3.54) | 4.15  (0.70,24.69) | 0.58  (0.12,2.79) |  |  |  |  |  |
| **auricular acupressure + TEAS** | 1.79  (0.73,4.40) | 0.62  (0.20,1.94) | 0.40 (0.12,1.33) | 1.92  (0.46,7.99) | **0.27**  **(0.08,0.95)** | 0.46  (0.09,2.44) |  |  |  |  |
| **auricular acupuncture** | 2.66  (0.22,31.93) | **0.92**  **(0.07,12.23)** | 0.59 (0.04,8.09) | 2.86 (0.19,43.44) | **0.40**  **(0.03,5.61)** | **0.69**  **(0.04,11.98)** | 1.49  (0.11,20.94) |  |  |  |
| **body and auricular acupressure** | 13.52  (0.63,291.83) | 4.70  (0.20,109.85) | **3.02**  **(0.13,72.24)** | 14.57  (0.56,380.94) | **2.04**  **(0.08,49.85)** | 3.51 (0.12,102.81) | 7.57 (0.31,185.98) | 5.09 (0.10,264.80) |  |  |
| **auricular electroacupuncture + auricular acupressure** | **3.31**  **(1.39,7.89)** | 1.15 (0.60,2.22) | 0.74  (0.30,1.80) | 3.57  (0.88,14.52) | 0.50 (0.16,1.53) | 0.86 (0.29,2.59) | 1.85 (0.53,6.43) | **1.25 (0.09,17.35)** | 0.24 (0.01,5.96) | 0.33 (0.01, 7.63) |

**End of Part 6**.

**End of Supplementary File1**.
